# Laser Speckle Imaging-Based Data-Driven Sensor for Comprehensive Monitoring of Drought Stress in Soybean Plants

**DOI:** 10.3390/s26216906

**Published:** 2026-10-31

**Authors:** Puneet Singh Thakur, Parama Pal, Beena Rai, Laxman Singh Rajput, Subhash Chandra, Vimal Bhatia, Ondrej Krejcar, Robert Frischer, Shashi Prakash

**Affiliations:** 1 TCS Research, Bangalore 560066, India; puneets.thakur@tcs.com (P.S.T.); beena64@gmail.com (B.R.); 2 Shell India Markets Ltd., Bangalore 562149, India; parama.pal@gmail.com; 3 ICAR- Indian Institute of Soybean Research, Indore 452001, India; laxman0742@gmail.com (L.S.R.); chandraasubhash@gmail.com (S.C.); 4 Division of Plant Improvement and Pest Management, ICAR- Central Arid Zone Research Institute, Jodhpur 342003, India; 5 ICAR-Central Institute for Cotton Research, Regional Station, Sirsa 125055, India; 6 Department of Electrical Engineering and Centre for Advanced Electronics, IIT Indore, Indore 452020, India; 7 Research Center, Skoda Auto University, Na Karmeli 1457, 29301 Mlada Boleslav, Czech Republic; robert.frischer@uhk.cz; 8 Faculty of Informatics and Management, University of Hradec Kralove, 50003 Hradec Kralove, Czech Republic; 9 Photonics Laboratory, Department of Electronics & Instrumentation Engineering, Institute of Engineering & Technology, Devi Ahilya University, Khandwa Road, Indore 452001, India; sprakash@ietdavv.edu.in

**Keywords:** agricultural productivity, drought stress, laser speckle imaging (LSI), machine learning (ML), photonics sensing

## Abstract

Identification and timely management of drought stress is a major challenge for global food security and sustainable agriculture. Existing techniques for drought identification are indirect, complex, and time consuming and require sophisticated equipment. Therefore, in this work, we present a novel optical sensor based on laser speckle imaging (LSI) integrated with machine learning (ML) for early identification of drought stress in soybean (*Glycine max*) plants. The objective of the study was to analyze the effectiveness of the proposed sensor in handling three key aspects of drought stress management: (i) detection of drought stress, (ii) early identification of stress symptoms, and (iii) discrimination of contrasting soybean genotypes with known differences in drought tolerance. Experiments were performed under controlled laboratory conditions, and speckle patterns were captured by the LSI sensor. Statistical feature extraction based on a 2D gray-level co-occurrence matrix (2D-GLCM) was employed, and various ML models were trained. XGBoost achieved the highest accuracy (96.67%), followed by random forest and decision tree (95.56%), for early identification and classification of drought in soybean plants. Moreover, the proposed sensor distinguished the two contrasting genotypes examined in this study (drought-tolerant JS 97-52 and drought-susceptible JS 95-60) with an accuracy of 95.83%, indicating that speckle-derived features are sensitive to their differing drought responses. By combining LSI-based photonics sensing with data-driven modelling, this work introduces an efficient solution for drought stress monitoring under controlled conditions, with potential for precision agriculture following further field validation.

## 1. Introduction

Drought stress is a major limiting factor that significantly hampers the growth and productivity of various crops worldwide [1]. It occurs because of insufficient soil moisture due to a lack of adequate irrigation and/or rainfall. It is estimated that around 83% of the total harvested area for coarse cereals, 80% for pulses, and 73% for oilseeds are under rainfed conditions [2]. However, in recent years, global warming and other climate changes have fueled a surge in both the frequency and duration of droughts. These changes have caused a substantial reduction (approximately 9–10%) in global crop yields [1]. Drought conditions significantly hamper various physiological and biochemical processes within plants such as water uptake, photosynthesis, and nutrient absorption. This inhibits plant growth and results in decreased leaf area, impaired pod development, reduced yield, and a multitude of other detrimental outcomes. 

Soybean (*Glycine max*) is a versatile legume crop that plays a crucial role in global agriculture, serving as a valuable source of plant protein, oil, and food [3]. It is a major oilseed crop produced and consumed worldwide and also holds immense economic significance in India. However, the growth and productivity of soybean plants are highly susceptible to drought stress, as they require considerable amounts of water to produce healthy crops. Drought stress interrupts various essential physiological and biochemical processes related to germination in the soybean. Consequently, the quality of soybean crops is significantly compromised, and a reduction in growth is observed at all developmental stages. More than 90% of India’s top oilseed crop is cultivated in rainfed conditions, where drought stress commonly occurs at various stages throughout the cropping season [4]. As a consequence, severe and prolonged drought stress can lead to irreversible damage, resulting in significant yield losses up to 80% depending upon the plant growth stage. Therefore, timely identification and accurate monitoring of drought stress enable farmers to implement proactive measures, adjust irrigation practices, and minimize yield losses. By comprehending the effects of drought stress on soybean plants, farmers can take timely action to mitigate its impact and enhance crop resilience. 

The management of drought conditions in plants comprises various objectives to mitigate the negative impacts of water scarcity on plant growth and, consequently, in agricultural production. There are three major components to be analyzed for efficient drought stress monitoring for optimal resource utilization and reduction in yield loss. These components comprise (i) the identification and classification of drought stress severity, (ii) early detection of drought symptoms, and (iii) identification of drought-tolerant varieties. Understanding the signs and symptoms of drought stress is imperative for timely intervention of corrective measures and effective management. Detecting drought stress at its early stages allows for the incorporation of corrective measures promptly for better crop protection. The proactive approach enables the implementation of needed interventions as per the growth stage of the plants to minimize yield losses. Moreover, understanding the inherent genetic tolerance mechanism of a specific variety towards drought stress allows breeders to select potential plants for the development of better soybean cultivars. Hence, there is a need for technological intervention that can comprehensively address all three aforementioned objectives to effectively manage drought stress and can improve the global food supply chain.

Traditionally, destructive analysis of water stress in plants is performed by measuring relative water content (RWC) in terms of leaf water status, stem water potential, electrolyte leakage, and stomatal conductance [1,2,3]. Moreover, other methods for drought testing and irrigation planning mainly focus on the direct measurement of various physiological parameters, such as soil moisture estimation, plant water potential measurement, and observing the morphology of plants [3]. These measurements are considered to be effective indicators of water stress in plants; however, these traditional measures are slow, manual and destructive, labor intensive, possess complex procedure, involve expert and trained personnel, and are not suitable for real-time identification of water stress. Optical methods also play a crucial role in the identification of drought stress in plants. These methods utilize various aspects of light interaction with plant tissues and provide valuable insights into the physiological and morphological changes induced by drought. Various optical methods including hyperspectral imaging (HSI), multispectral imaging [5], dynamic laser speckle imaging [6], near-infrared spectroscopy (NIRS), infrared thermography, and fluorescence imaging have been used for the identification and classification of drought stress in plants [7]. These methods can perform monitoring of biochemical changes related to drought stress in plants. Spectral imaging techniques excel in capturing critical physiological changes in plants and provide direct assessment of the plant’s water status. The notable advantages of these optical methods are non-destructive measurements, spatial visualization, the ability to extract metabolic insights, detailed spectral information for comprehensive analysis, and automatic operation. However, these methods also possess various drawbacks, including sensitivity towards external factors (such as illumination conditions and vibration) and environmental perturbation [8]. Additionally, these methods also suffer from complexities involved with experimental setup, calibration procedures, data acquisition, image processing, and the requirement of expensive and specialized high-quality optical components. 

In recent years, computer vision-based methods have become increasingly important for the identification and assessment of drought stress in plants [9]. This interest is primarily attributed to the integration of machine learning (ML) and deep learning (DL) algorithms, which allow for automatic extraction of minute and specific features from RGB images. Consequently, these techniques can perform qualitative assessment of various drought-related parameters (morphological (i.e., shape, size, color, and texture), spectral, and temporal variations) [10]. These existing machine vision-based techniques primarily focus on visible symptoms of drought stress appearing on the plant’s surface. However, drought stress results in various biological changes inside plants, including alterations in internal anatomical structures, biological processes, and physiological characteristics [10]. Therefore, accurate identification of drought stress is not possible by just analyzing the external morphology of the plants. These vision-based techniques overlook critical biological aspects during evaluation. Consequently, these methods frequently exhibit several limitations related to repeatability, robustness, and accuracy in prediction. As these methods rely mainly on surface appearance, their ability to detect drought stress before visible symptoms appear, or to evaluate the drought tolerance of different varieties, may be limited. Furthermore, all indirect methods are usually applied during physiological maturity of the plants and therefore require an entire growing season and extended experiment duration. 

Considering these limitations, there remains a need for a more comprehensive and biologically informed sensor that is non-invasive, fast, and capable of monitoring drought under practical conditions. In this direction, laser speckle imaging (LSI) offers a new approach by leveraging coherent light interactions to capture dynamic physiological changes in plant tissues. Unlike traditional destructive methods or complex spectral imaging systems, LSI provides a simple yet useful form of optical sensing that is highly sensitive to microstructural and metabolic changes in plants induced by drought stress. Due to its rapid and non-contact measurements with minimal complexity in setup, LSI has become a promising solution for next-generation optical sensors for different agricultural applications. Via integration with advanced ML-based algorithms, this sensing approach enables early detection, severity assessment, and genetic tolerance characterization, thereby addressing critical gaps in current drought stress monitoring practices.

In this work, we have developed an LSI-based intelligent sensor to analyze various aspects of drought stress management in soybean plants. The main contributions of the research given are as follows:

- We developed a novel optical sensor based on laser speckle imaging (LSI) for non-invasive identification and classification of drought stress and its severity in a timely manner. Moreover, the proposed sensor enables early-stage detection and quantitative measurement of drought symptoms in plants, enabling proactive management strategies for sustainable agriculture.
- Characterization of the drought tolerance capability of given plant varieties was also performed to discriminate the two contrasting genotypes for efficient and economic drought management.
- We designed an end-to-end ML-based photonics sensor for identifying drought stress in soybean plants using speckle images. This pipeline employs a GLCM-based feature extraction approach to analyze the spatial variations in speckle images.
- Comprehensive evaluation of various ML models was undertaken to classify drought stress in soybean plants. The proposed approach achieved higher accuracy for classification of drought stress, early identification of drought symptoms, and classification of drought-tolerant varieties.

The rest of the paper is organized as follows: Section 2 contains a description of the materials and methods used for this study. The subsections in this segment provide details of the sample preparation followed by a description of the experimental assembly for the acquisition of speckle images. Section 3 provides a comprehensive description of image processing techniques used for feature extraction and ML architecture for classification of data. Section 4 presents a detailed analysis of the results obtained from the study. Finally, Section 5 summarizes the principal contributions of the research article.

## 2. Materials and Methods

### 2.1. Experimental Design and Plant Material

In this section, we outline the experiments conducted to create the speckle dataset for three distinct objectives related to drought stress monitoring and management in soybean plants. Morphological and physiological parameters were also measured to validate the feasibility and biological relevance of the proposed framework. Five diverse and widely cultivated soybean varieties from the Indian seed chain were used, namely JS 97-52, JS 20-69, JS 20-98, Bragg, and JS 95-60, including the drought-tolerant genotype JS 97-52 and the drought-susceptible genotype JS 95-60. Experiments were conducted under controlled greenhouse conditions at ICAR-Indian Institute of Soybean Research (IISR), Indore, Madhya Pradesh, India, with daytime and nighttime temperatures maintained at 25 ± 2 °C and 18 ± 2 °C, respectively. For each genotype, two independent experimental groups of plants were grown. In each group, there were 20 pots containing 3 plants per pot, i.e., 60 plants per genotype per group. Across the five genotypes, each group therefore comprised 300 plants, giving 600 plants in total for Experiment 1. The first group generated the optimum moisture class, while the second independent group was used to generate both the early-stress (days 15–20) and stressed (days 20–25) classes. Consequently, optimum moisture observations originated from a completely independent set of plants, whereas early-stress and stressed observations were acquired from the same plants monitored at successive stages of drought progression.

Laser speckle measurements were carried out only on fully expanded trifoliate leaves collected from comparable nodal positions and similar developmental stages across different plants within a given stress condition. To ensure consistency and biological comparability, measurements were performed prior to severe tissue degradation, and leaves showing advanced senescence, necrosis, or structural collapse were excluded from optical analysis. In soybean plants, trifoliate leaves at the same node are widely recognized to exhibit similar anatomical structure, thickness, chlorophyll content, and physiological maturity, making them suitable for controlled physiological comparisons [11]. As detailed in the preceding section, the study aimed to explore the potential of laser speckle imaging (LSI) in conjunction with machine learning to address three important objectives of drought stress monitoring and management in soybean plants. To accomplish this, two distinct experiments were carried out as described below.

**Figure 1:**
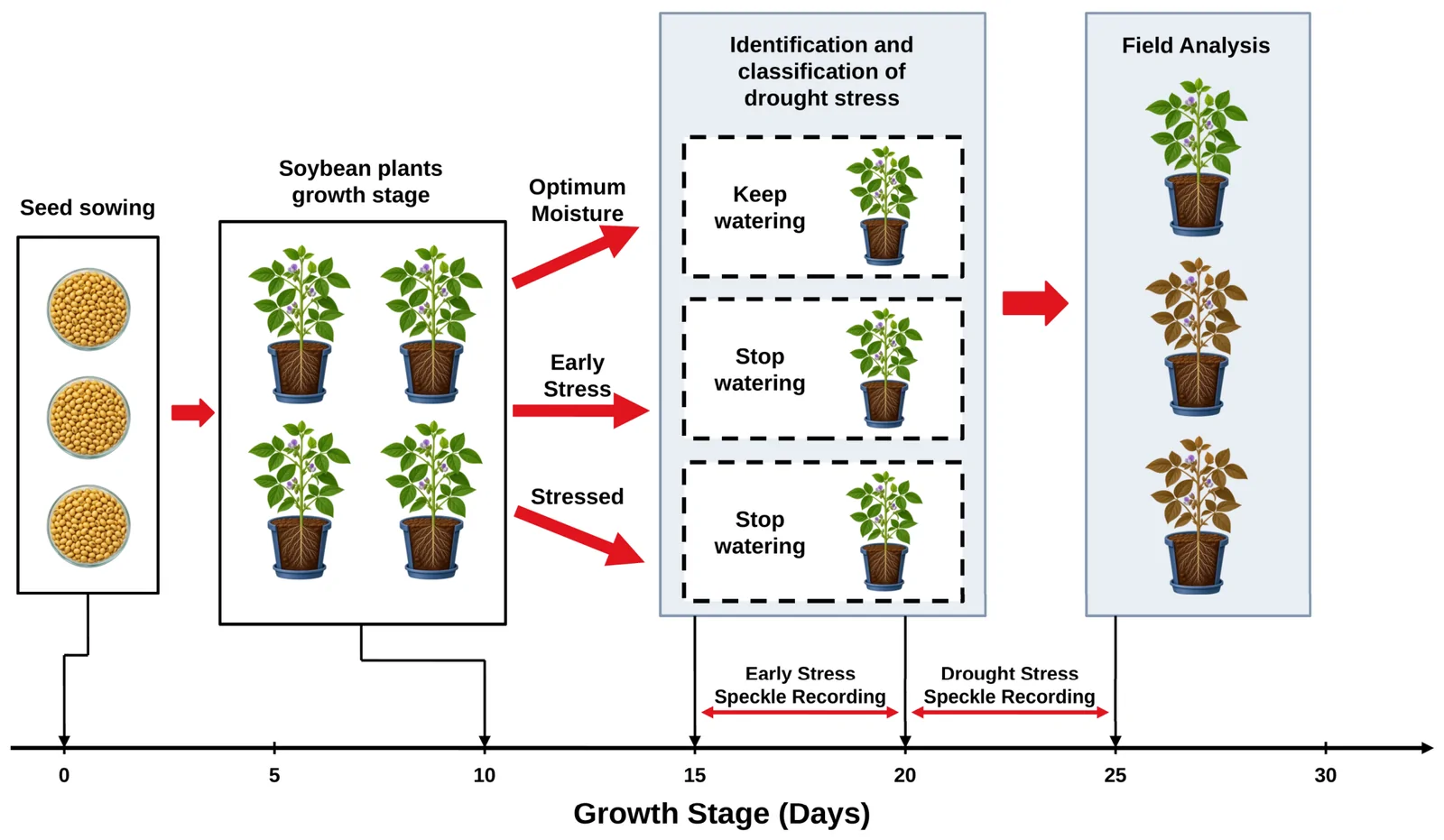
Description of the protocols for monitoring drought stress during growth stages. Arrows indicate the sequence of experimental stages and the speckle recording windows (days 15–20 for early stress and days 20–25 for stressed).

Experiment 1. Classification of drought stress severity and early-stage identification: The first two objectives of the study were to identify drought stress in plants and characterize its symptoms in early stages. Therefore, in this experiment, the plants from two independent groups were subjected to different watering conditions to create three different classes of datasets, namely optimum moisture, early stress, and stressed (as shown in Figure 1).

To start the experiment, two sets of plant groups (20 pots per variety with 3 plants in each pot, i.e., 60 plants per genotype) with homogenous conditions were grown. The individual plant was treated as the biological unit of observation and data partitioning throughout this study, whereas the pot, in which three plants shared the same soil and watering regime, was the unit to which the watering treatment was applied. Plants from the first group (Group 1) were used as a control sample (optimum moisture), while in the second group (Group 2), a separate set of 60 plants per genotype (300 plants in total) was used to generate both the early-stress and stressed classes at successive time points during drought progression. Before dataset partitioning, every feature vector derived from these plants was linked to a unique biological identifier comprising the genotype, pot number, and plant number, ensuring that all observations from a given plant could be tracked consistently across classes and downstream analysis. Ten absolutely healthy seeds with uniform shape and size were sown at a 4 cm depth in plastic pots (45 cm height and 18 cm diameter) inside a soil and farmyard manure mixture (ratio: 2:1). After two weeks of emergence, thinning of the plants was performed, and only the three most vigorous plants per pot were allowed to grow. At the initial stage, all pots were nurtured with irrigation to obtain soil at field capacity. To create the dataset for optimum moisture class, speckle images were recorded for all the varieties of soybean from day 15 till day 25, later sowing with continuous watering (Figure 1). For each plant, 10 speckle images were recorded per condition, giving 3000 speckle images per class (300 plants × 10). The images of all five genotypes were pooled within each class. However, after 15 days of sowing, drought stress was induced in the plants by withholding irrigation to create the two other classes. In pots, soil moisture was recorded through gravimetric methods in both sufficient moisture and drought conditions to identify actual moisture stress conditions in the soil. For the early-stress class, a second group of plants was chosen, and speckle images were captured from the 15th to the 20th day after sowing, during a period when visual symptoms were not apparent (Figure 1). Similarly, for the stressed group, data were collected from the 20th to the 25th day, when plants exhibited visible signs of drought stress.

To validate the feasibility of the study, we also recorded various morphological and physiological parameters for the aforementioned plant samples. Following the completion of speckle data acquisition, a selected subset of these plants (30 plants per genotype in each group, i.e., 10 pots × 3 plants; 300 plants in total) was utilized to measure plant dry weight (PDW), relative water content (RWC), and SCMRs (SPAD chlorophyll meter readings) under both optimum moisture and moisture deficit conditions [12]. The same subset was used for the correlation analysis in Section 4.2. These measurements collectively provide a comprehensive view of plant stress levels, considering both the morphological aspects (dry weights) and physiological responses (RWC and chlorophyll content). PDW (typically measured in grams per plant) refers to the biomass of dried plants (in oven at 70 °C for 72 h) after completion of the experiment (i.e., after 25 days). A decrease in PDW often indicates stress conditions, which negatively impacts the photosynthetic efficiency and other metabolic processes in the plants. This, in turn, hinders the plant’s ability to accumulate biomass. RWC is a measure of water status within plant tissues. It is expressed as a percentage and quantifies the amount of water present in the plant at a particular stage relative to its maximum water-holding capacity. SCMR values serve as a measure of chlorophyll content and can provide insights about water stress conditions in plants. 

Experiment 2. Identification of drought tolerance of given soybean varieties in the early stage: The next experiment was planned to examine the status of genetic tolerance of soybean varieties towards drought stress using laser speckle imaging. Before starting these experiments, screening for drought tolerance among the selected soybean varieties was carried out in field conditions. Based on this preliminary evaluation, two contrasting soybean genotypes, namely drought-tolerant JS 97-52 and drought-susceptible JS 95-60, were selected for further investigation. The use of contrasting genotypes is a widely adopted approach in plant stress physiology and phenotyping studies, as it enables sensing methodologies to be evaluated across distinct physiological response patterns under controlled conditions [4]. These genotypes exhibit contrasting responses to water-deficit stress in terms of hydration status, chlorophyll retention, and stress adaptation behavior, thereby providing an appropriate framework for assessing the capability of LSI to detect drought-induced physiological variations.

For this, parameters defining genetic variation in drought tolerance at reproductive stages for all soybean varieties were calculated. To analyze this, all soybean varieties were grown in field conditions under a randomized block design in a rainout shelter with standardized protocols [12]. After providing drought stress at the reproductive stage (from the 45th day until the harvesting stage) inside the rainout shelter, various seed-related parameters, including drought tolerance indices (DTIy), percentage reduction in 100-seed weight (Rsw (%)), and percentage change in plant dry weight ((g)/plant (%Rpdw), were calculated for both tolerant and susceptible varieties at the end of the experiment. All of these parameters are presented in a subsequent section of the manuscript. 

**Figure 2:**
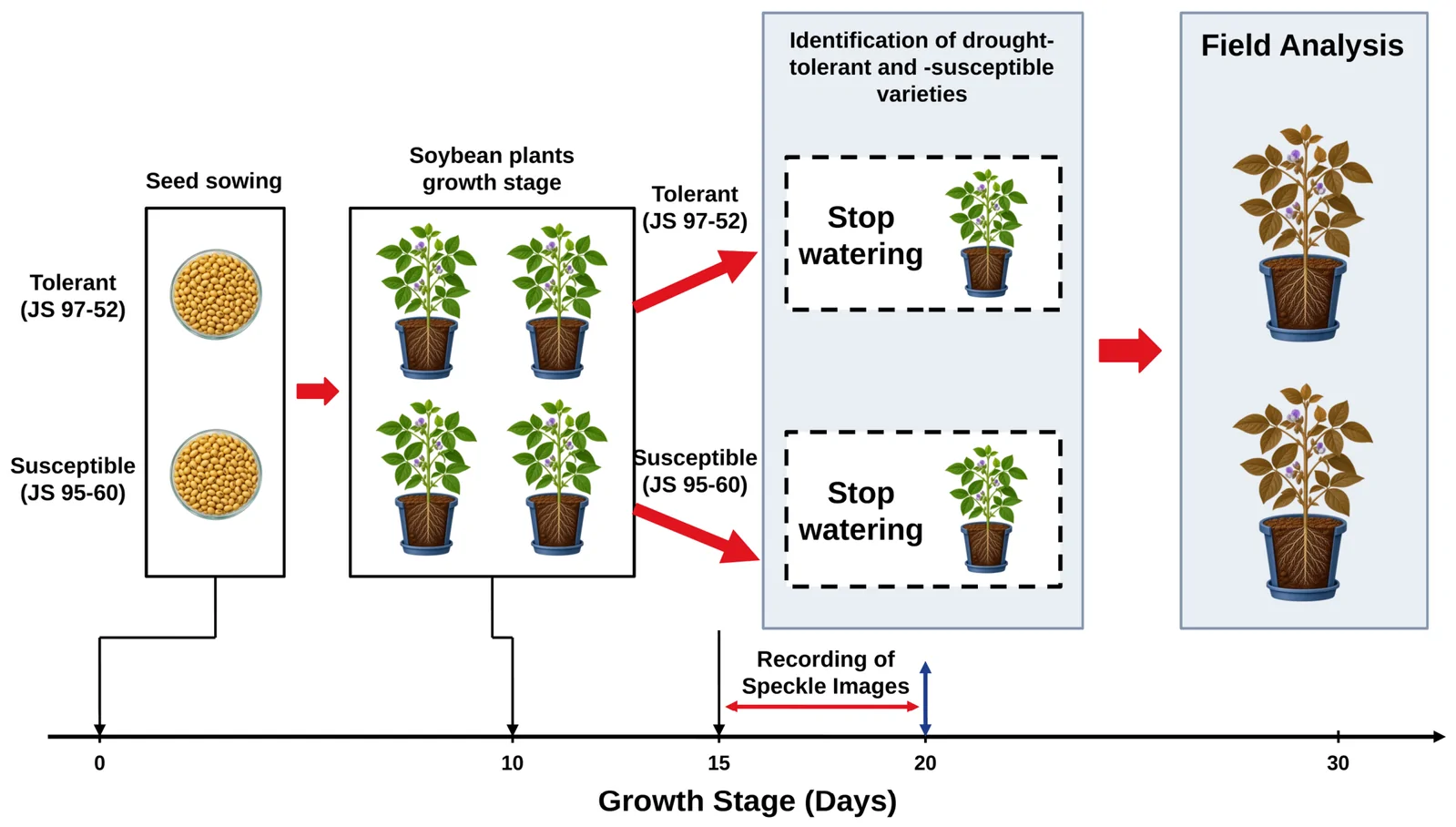
Description of the protocols for monitoring drought tolerance of different varieties during growth stages (Arrows indicate the sequence of experimental stages and the speckle recording window (days 15–20)).

By utilizing the data from the above experiments, two soybean varieties (with known tolerance towards drought), JS 95-60 (susceptible) and JS 97-52 (tolerant), were selected and grown to record speckle data. A description of the experimental protocols is visually presented in Figure 2. In this experiment, two plant groups (30 pots per variety with 3 plants in each pot, i.e., 90 plants per variety and 180 plants in total) were grown under identical conditions. The plants from both classes were watered continually for 15 days from sowing with the recommended amount of water. After this stage, the watering of both plant groups was stopped, and continuous recording of speckle images was performed for the stress period of 5 days (from 15th to 20th days) for analyzing the drought tolerance capability of the given soybean varieties in the early stages. From each plant, 20 speckle images were recorded, giving 1800 speckle images per class.

### 2.2. Statistical Analysis

The obtained morpho-physiological parameters were statistically analyzed using the “Agricolae” package within R Studio software version R 4.2.3 (R Core Team 2017). Morpho-physiological and other traits were recorded from the plants of each selected pot as per standard protocol and averaged for an experimental unit (the pot, used as the experimental unit for ANOVA and LSD). This included analysis of variance (ANOVA) and multiple comparison tests employing the least significant difference (LSD) method. The main hypothesis for this analysis was to analyze the possible effect of drought on morpho-physiological parameters of soybean genotypes. Differences among the means for all measurement events were evaluated for significance using the LSD approach.

### 2.3. Laser Speckle Imaging

Laser speckle imaging (LSI) [11] is employed as the core sensing technology in this study, offering a non-invasive optical approach to characterize drought-induced physiological changes in plants. LSI operates by capturing speckle patterns generated due to mutual interference of different coherent wavefronts reflected from the surface of a sample under study. When a coherent light source (such as a laser) illuminates the surface of a given sample, each point of reflection serves as a secondary source of wavefronts, resulting in a statistical interference pattern termed “speckle”. LSI can effectively capture various biological changes in samples and inherently possesses several advantages such as non-destructive operation, easy alignment, simple experimental procedures, lower memory requirements, and less complex image acquisition and processing.

**Figure 3:**
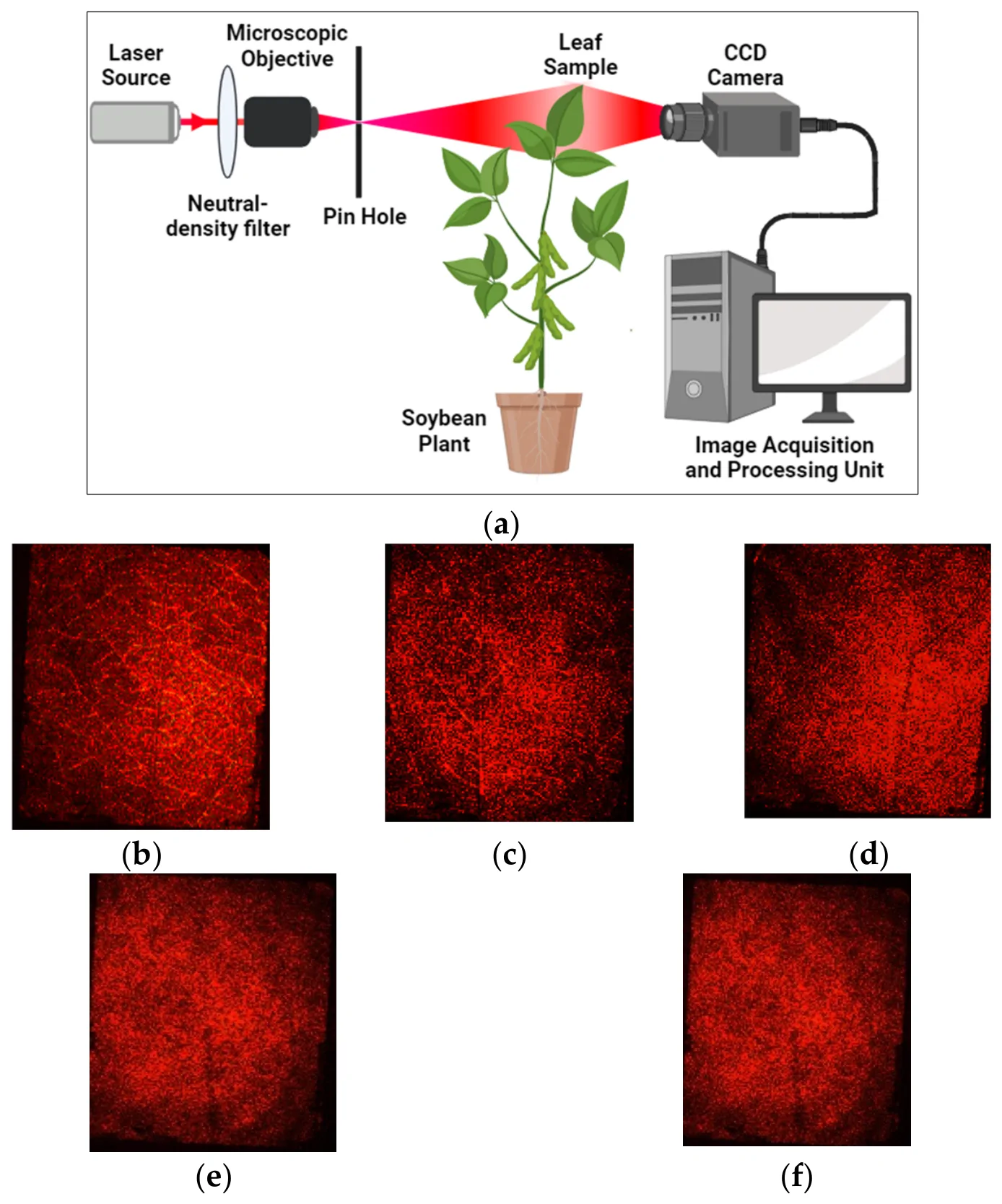
(**a**) Experimental setup used for acquisition of speckle images; speckle images for (**b**) optimum moisture content, (**c**) early-stressed, and (**d**) stressed plant leaves; (**e**) drought-susceptible and (**f**) drought-tolerant genotypes.

The sensing system was implemented using a custom-designed experimental setup optimized for the acquisition of high-quality speckle images. A detailed sensor setup, used to capture speckle images from leaf samples, is illustrated in Figure 3a. The measurements were carried out using a standard coherent optical speckle setup under controlled laboratory conditions, and all measurements were performed on a vibration-isolated optical table under controlled lighting conditions to minimize external disturbances. The optical system consisted of a Helium–Neon (He–Ne) laser source (output power 15 mW; wavelength 632.8 nm), a variable attenuator, a spatial filtering and beam-conditioning assembly, a CCD camera, and a computer for image acquisition and processing. The laser beam intensity was adjusted using a variable neutral-density filter (Thorlabs, USA) to control the optical power incident on the sample and mitigate the possibility of under- or over-exposure. Spatial filtering was performed using a microscope objective (40× magnification) in combination with a pinhole of 10 µm diameter positioned at a distance of 80 mm from the laser source. This spatial filtering arrangement removed non-uniformities in the laser profile and produced a clean and uniformly expanded beam. The filtered beam was then collimated using a precision achromatic doublet lens with a focal length of 250 mm and a diameter of 50 mm. Speckle patterns generated after interaction with the sample were recorded using a digital RGB CCD camera (Basler Corp., Germany) with a resolution of 1024 × 967 pixels, a frame rate of 32 fps, and a pixel size of 3.45 × 3.45 µm. The camera was positioned approximately 150 mm from the sample plane and oriented perpendicular to the direction of illumination. The average speckle size at the detector plane was adjusted to approximately 10–15 µm, corresponding to about 2–3 camera pixels per speckle, thereby satisfying standard speckle sampling and Nyquist criteria. Single-frame speckle images were recorded with exposure times in the range of 5–10 ms, avoiding temporal averaging of speckle fluctuations while preserving spatial speckle statistics and maintaining an adequate signal-to-noise ratio. 

In this study, a tabletop experimental assembly was created in a transmission geometry and integrated with a specially designed 3D-printed leaf holder. The collimated beam was subsequently allowed to pass through the leaf sample in a transmission-based configuration. This penetration-based geometry ensured that the coherent illumination interacted with the internal leaf microstructure, enhancing sensitivity to hydration- and structure-dependent scattering changes. The custom-designed leaf holder served the dual purpose of maintaining sample stability and capturing precise leaf regions to yield high-quality and repeatable speckle measurements. Leaf samples were mounted on a rigid fixed holder to maintain stable positioning and consistent imaging geometry throughout image acquisition. After optimization of the optical and imaging parameters, speckle patterns were captured for different plant samples. Representative speckle images corresponding to optimum moisture, early-stress, and stressed conditions are presented in Figure 3b, Figure 3c, and Figure 3d, respectively.

## 3. Image Processing Strategy

Speckle images were recorded using the proposed sensor for all five soybean varieties under the different experiments described in the previous section. For the first two objectives, the dataset consisted of three classes, namely optimum moisture, early stress, and stressed, with each class containing 3000 speckle images. Similarly, for the identification of genetic tolerance in soybean varieties, two classes (tolerant and susceptible) were created, each containing 1800 speckle images.

Although speckle images were collected from multiple plants under the same experimental condition within a given class on successive days during the drought progression experiment, the raw images were not treated as independent samples for machine learning analysis. Instead, GLCM features were first computed for individual speckle images, and subsequently, groups of 10 speckle images acquired under identical experimental conditions were averaged to generate a single feature vector representing physiological observation. This aggregation procedure served two important purposes: (i) it reduced stochastic speckle variability inherent to coherent imaging systems, and (ii) it suppressed frame-level temporal correlation between sequential images.

To obtain statistically stable descriptors, GLCM features were averaged over groups of 10 speckle images, yielding a single feature vector representing a given physiological state. This procedure provided 300 feature vectors per class for Experiment 1 (one per plant for each condition; 900 in total) and 180 feature vectors per class for Experiment 2 (two per plant; 360 in total). Each feature vector was computed from images of a single plant. Consequently, the final dataset used for machine learning consisted of feature-level observations corresponding to physiological states (optimum moisture, early stress, and stressed), rather than individual sequential frames of the same plant. Before partitioning, every feature vector was linked to a unique biological identifier comprising the genotype, pot number, and plant number. Plants were assigned exclusively to the training, validation, or testing subset, and all feature vectors originating from a given plant were retained within the same subset. Biological independence was therefore maintained through plant-exclusive partitioning, ensuring that no individual plant was represented in more than one subset. Acquisition-session identifiers were evaluated separately during dataset construction. The dataset split (60% training, 20% validation, and 20% testing) was then applied at the plant level. In Experiment 1, the 600 plants were assigned as 360, 120, and 120 unique plants to the training, validation, and independent test sets (Groups 1 and 2 each split 180/60/60), corresponding to 180, 60, and 60 feature vectors per class. In Experiment 2, the 180 plants were assigned as 108, 36, and 36 plants (54, 18, and 18 per genotype), corresponding to 108, 36, and 36 feature vectors per class. Zero plant overlap among the three subsets was confirmed in both experiments. In addition, stratified group-based 10-fold cross-validation was employed during model evaluation using plant identity as the grouping variable, further reducing the possibility that model performance depended on a particular data partition. The fold-wise cross-validation results are reported in Section 4.3. It is also important to note that the objective of this study was the classification of drought stress states rather than time-series prediction of individual plants. Therefore, the machine learning models learned condition-dependent speckle texture signatures associated with physiological drought stress rather than exploiting temporal continuity between successive measurements. Accordingly, the analysis performed in this study does not treat the data as a time-series problem, and specific measures were implemented to avoid information leakage and overoptimistic performance estimation.

**Figure 4:**
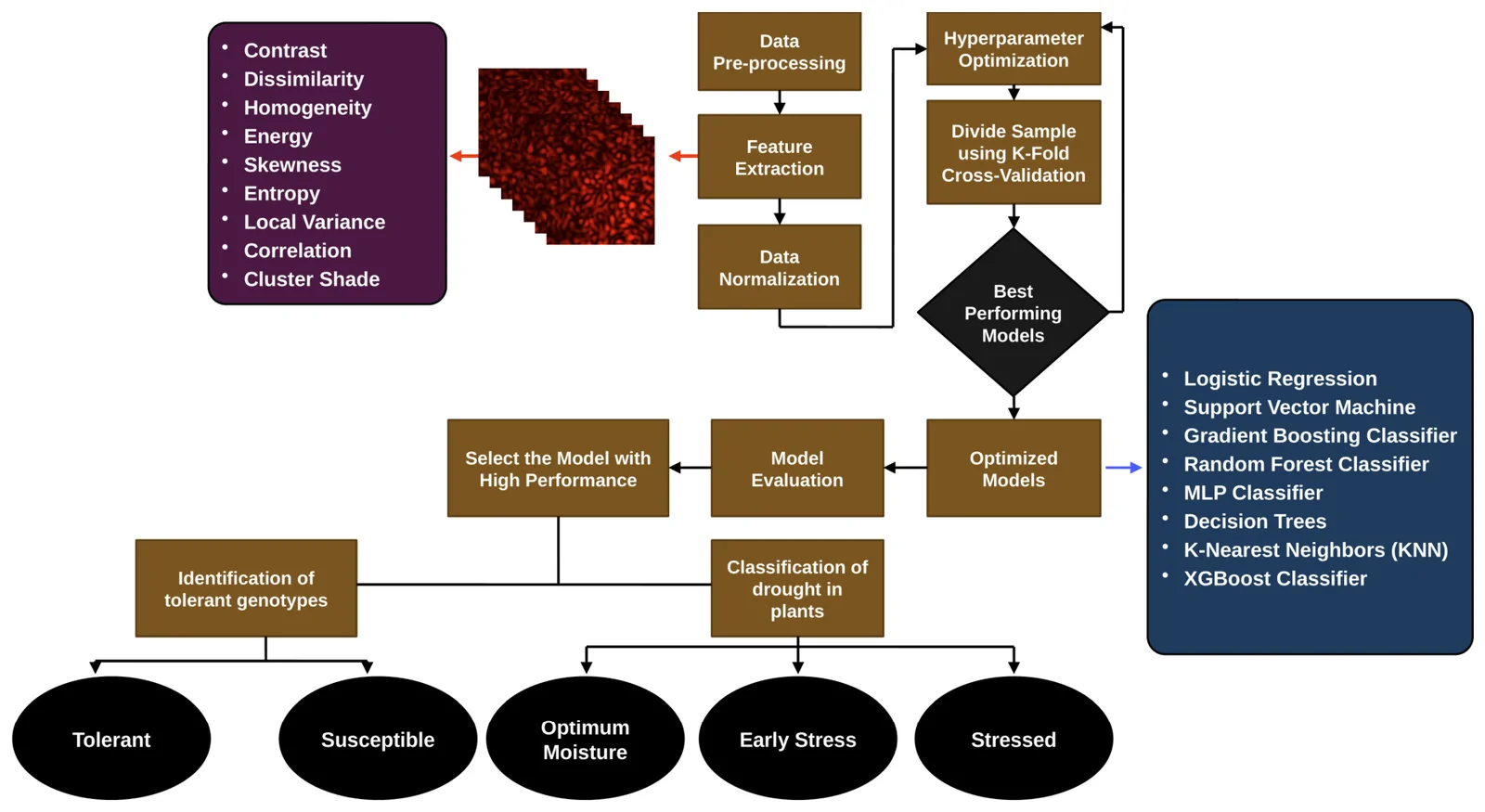
Overall image processing framework for feature extraction and classification of speckle data, showing the nine extracted GLCM texture descriptors, the evaluated machine learning classifiers, and the two classification tasks (drought tolerance identification and drought stress classification).

Description of the dataset is given in Table 1 and Table 2. Figure 4 presents a flow diagram of the image processing framework utilized for the identification and classification of drought stress. The image processing strategy consists of different stages, including data pre-processing and feature extraction, development of the ML model, and evaluation of the developed model for given classification tasks. In this work, acquired speckle images are processed by extracting features from pre-processed speckle images by using a gray-level co-occurrence matrix (GLCM) [13]. Furthermore, these features are utilized to train and test various ML models for classification tasks. An explanation of the steps involved in the processing of speckle data is provided in the subsequent sections.

### 3.1. Image Pre-Processing and Feature Extraction

Figure 4 shows the flow diagram of the steps involved in the processing of speckle images and the development of the ML model for the classification task. The acquired speckle images are first cropped to a standardized dimension of 300 × 300 pixels to achieve uniformity in image size and facilitate efficient processing. Furthermore, color space conversion to grayscale was performed, followed by feature extraction. The color conversion not only reduced data dimensionality but also allowed for a simplified and consistent representation of the images, which is particularly advantageous for further analysis.

These pre-processed images are further used for feature extraction by using a GLCM. GLCM-based feature extraction is a well-established and widely accepted technique for laser speckle image analysis, which exploits the relationship between neighboring pixels spaced by a specific distance in an image. These features possess rapidly changing pixel values for fine-texture regions and a slowly changing value in the coarse-texture regions. GLCM-based analysis offers a statistical characterization of the spatial relationships between pixel intensity values in speckle images and represents the second-order statistical relationship between image pixels. Laser speckle patterns arise from coherent interference processes that are governed by second-order intensity correlations rather than absolute intensity values. Consequently, the GLCM provides a physically meaningful framework for extracting relevant texture features from speckle images. This allows for a more comprehensive understanding of the textural properties of the speckle pattern. By quantifying the frequency of occurrence of different pixel intensity pairs at specific spatial relationships, GLCM-based features capture fine-grained details and subtle variations within the speckle image.

A total of nine texture descriptors were extracted from the speckle images, including six second-order GLCM descriptors (contrast, dissimilarity, energy, homogeneity, correlation, and entropy), one higher-order GLCM descriptor (cluster shade), and two complementary statistical descriptors (skewness and local variance). These descriptors collectively characterize spatial intensity variation, texture heterogeneity, local structural variation, and higher-order statistical properties associated with drought-induced changes in leaf tissue microstructure. Gray-level co-occurrence matrices (GLCMs) were computed from pre-processed grayscale speckle images using 256 gray-level quantization levels (Ng = 256), an inter-pixel distance of 1 pixel (d = 1), and four angular orientations (0°, 45°, 90°, and 135°). Symmetric and probability-normalized co-occurrence matrices were generated and averaged across all four directions to obtain rotation-invariant texture descriptors. To suppress stochastic frame-to-frame fluctuations inherent to coherent speckle imaging, descriptors extracted from groups of 10 images acquired under identical experimental conditions were averaged to generate a single feature vector for subsequent machine learning analysis. Cluster shade quantifies the asymmetry of the gray-level co-occurrence distribution and is sensitive to higher-order spatial intensity variations within the speckle pattern. Negative values indicate a negatively skewed co-occurrence distribution, whereas values closer to zero indicate reduced asymmetry in the underlying texture structure.

Consequently, texture descriptors computed from a single speckle frame can exhibit avoidable variance unrelated to drought-induced physiological changes. As mentioned in the previous section, 3000 speckle images were collected for each of the three classes in Experiment 1, and 1800 speckle images were collected for each of the two classes in Experiment 2. To enhance the robustness of model training and generalize speckle GLCM features, we calculated the average of GLCM features for 10 speckle images for each single observation. Consequently, the final dataset comprises 300 feature vectors per class for Experiment 1 and 180 feature vectors per class for Experiment 2. Finally, the obtained feature matrix is normalized in order to have features in the same range between 0 and 1. Data normalization not only improves the classification performance but also enhances the accuracy of the ML models. In this work, we utilized the “Min–Max” [14] technique to perform normalization of the data. This technique employed linear transformation on the original dataset and preserves the relationships among the original data while eliminating the bias.

(1) $$X_{norm}=\frac{X-X_{min}}{X_{max}-X_{min}}$$

Here, X represents the original feature value, and $X_{min}$ and $X_{max}$ denote the minimum and maximum values of the corresponding feature, respectively. This technique employs a linear transformation on the original dataset, preserves the relationships among the original data, and ensures balanced contribution of all descriptors during model training.

### 3.2. Permutation Feature Importance Analysis

Following feature normalization, permutation feature importance (PFI) was employed to identify the most discriminative GLCM features contributing to drought stress classification. The analysis provides insights into the relative importance of individual texture descriptors and their contribution towards model predictions. PFI is a model-agnostic feature interpretation method that quantifies the importance of a feature by measuring the decrease in model performance after randomly permuting the values of that feature while keeping all other features unchanged [14]. For a trained classifier, the importance of feature j was calculated as:

(2) $$I_{j}=Acc\left(D\right)-Acc(D_{\pi j})$$

where $I_{j}$ is the PFI, and $D_{\pi j}$ denotes the dataset in which feature j has been randomly permuted. A larger reduction in accuracy indicates greater importance of that feature for the prediction task. The permutation importance analysis was applied to the ensemble classifiers, namely random forest and XGBoost, to ensure that the interpretation reflects the behavior of the best-performing models rather than an arbitrary classifier. To ensure robustness, permutation importance was computed across stratified group-based 10-fold cross-validation, and mean importance values were used to obtain a stable ranking. The consistency of the feature ranking across folds indicates that the identified discriminative descriptors are not dependent on a particular training–testing split, thereby strengthening the reliability and replicability of the results.

### 3.3. Development of Machine Learning (ML) Model for Classification

All the acquired information associated with different classes was pooled into a file, and the observations were partitioned into training, validation, and testing sets at the plant level, using a unique biological identifier (genotype, pot number, and plant number) assigned to each feature vector, rather than by unconstrained random selection. This plant-exclusive partitioning ensured that all feature vectors originating from a given plant, including its contributions to more than one class, where applicable, were retained within the same subset, so that no individual plant was represented in more than one partition. The data partition ratio was set to 60%, 20%, and 20% for training, validation, and testing datasets, respectively. Some acquisition sessions were shared across partitions; therefore, biological independence was maintained at the plant level, while complete acquisition-session independence is not claimed. During the validation phase, the validation dataset was used for unbiased evaluation of predictive models while tuning the hyperparameters associated with each learning algorithm. Finally, the test dataset was used for evaluation of the final model generated from the training dataset.

To ensure optimal utilization of all the information residing in training data, evaluation of the trained/learned model is performed by using the k-fold cross-validation method [15]. In this method, all data points are divided into a defined number of subsets (say n), out of which n−1 subsets are put together to form a training set for the learning procedure of a model, and the remaining one is used for evaluation. The approach was repeated for all possible combinations of the evaluation set, and the average of all the possibilities was used to assess the performance of the generated model. In this study, stratified group-based 10-fold cross-validation was applied, with plant identity specified as the grouping variable, ensuring that observations from the same plant did not occur simultaneously in the training and held-out portions of any fold. Identical fold assignments generated using a fixed random seed (42) were applied across all classifiers. In Experiment 1, each fold contained approximately 90 feature vectors from about 60 plants; in Experiment 2, approximately 36 feature vectors from about 18 plants were used. In each iteration, Min–Max normalization and hyperparameter tuning used only the nine training folds, and the remaining fold was used exclusively for evaluation. For each classifier, the mean accuracy, standard deviation, and range across the ten folds were calculated and are reported in Section 4.3 together with the independent test-set accuracy. Because the cross-validation was run on all plants, it is a robustness check separate from the single plant-exclusive test-set evaluation. Various ML classifiers, including gradient boosting (GB) [16], random forest (RF) [17], XGBoost [18], decision trees (DT) [19], K-nearest neighbors (KNN) [20], support vector machine (SVM) [21], logistic regression (LR) [22], and multilayer perceptron (MLP) [23], were developed and compared in terms of their feasibility after optimizing their hyperparameters. All eight classifiers were evaluated in both experiments, and the six best-performing models for each classification task are reported in the Results.

While training the ML models, fine-tuning and optimization of hyperparameters were performed using a cross-validated grid-search algorithm [24]. Grid search is a widely used optimization technique that systematically evaluates all candidate combinations within a predefined hyperparameter space and selects the configuration yielding the highest validation performance. In this study, hyperparameter optimization was implemented using scikit-learn GridSearchCV class, which performs an exhaustive search over the specified parameter grid. The complete search space and final selected hyperparameter values for all classifiers are summarized in Table 3. Hyperparameter optimization was restricted to the training partition, and the validation partition was used for model selection. Min–Max normalization parameters were estimated from the training data only and subsequently applied to the validation and test data without refitting. Following the completion of the search procedure, the optimal parameter configuration for each classifier was used to train the final model, which was subsequently evaluated once on the independent test partition.

Finally, to measure the performance of the classification models, confusion matrices were generated [25]. A confusion matrix contains information related to the actual and predicted classifications performed by a learning algorithm. Model performance was evaluated using accuracy, specificity, precision, recall, and F1 score [25]. Furthermore, receiver operating characteristic (ROC) curves were generated to visualize classifier performance over a range of trade-offs between the true positive rate (TPR) and false positive rate (FPR) [25].

## 4. Results and Discussion

### 4.1. Identification and Classification of Drought Stress in Early Stages

To classify stressed plants and early-stage identification (before specific morphological features (such as leaves dropping, plant wilting, leaf yellowing, etc.) became visible), several ML algorithms were implemented, and the dataset is divided into three classes: optimum moisture, early stress, and stressed. The proposed models were developed using Python (version 3.10) with the scikit-learn (version 1.3.0) and SciPy (version 1.11.0) libraries, running on Windows 10 with an Intel i3 core processor and 8 GB RAM. A total of 300 feature vectors per class (900 in total, from 600 plants) were used to train, validate, and test the ML models, with an independent test set of 180 feature vectors (60 per class) from 120 plants. Table 4 summarizes the mean values and standard deviations of the nine extracted texture descriptors under the three drought conditions (optimum moisture, early stress, and stressed). These descriptors quantify the changes in spatial intensity distribution, texture heterogeneity, local structural variation, and higher-order statistical behavior of laser speckle patterns during drought progression. The reported values show how speckle-derived texture characteristics differ across the three drought conditions. 

**Figure 5:**
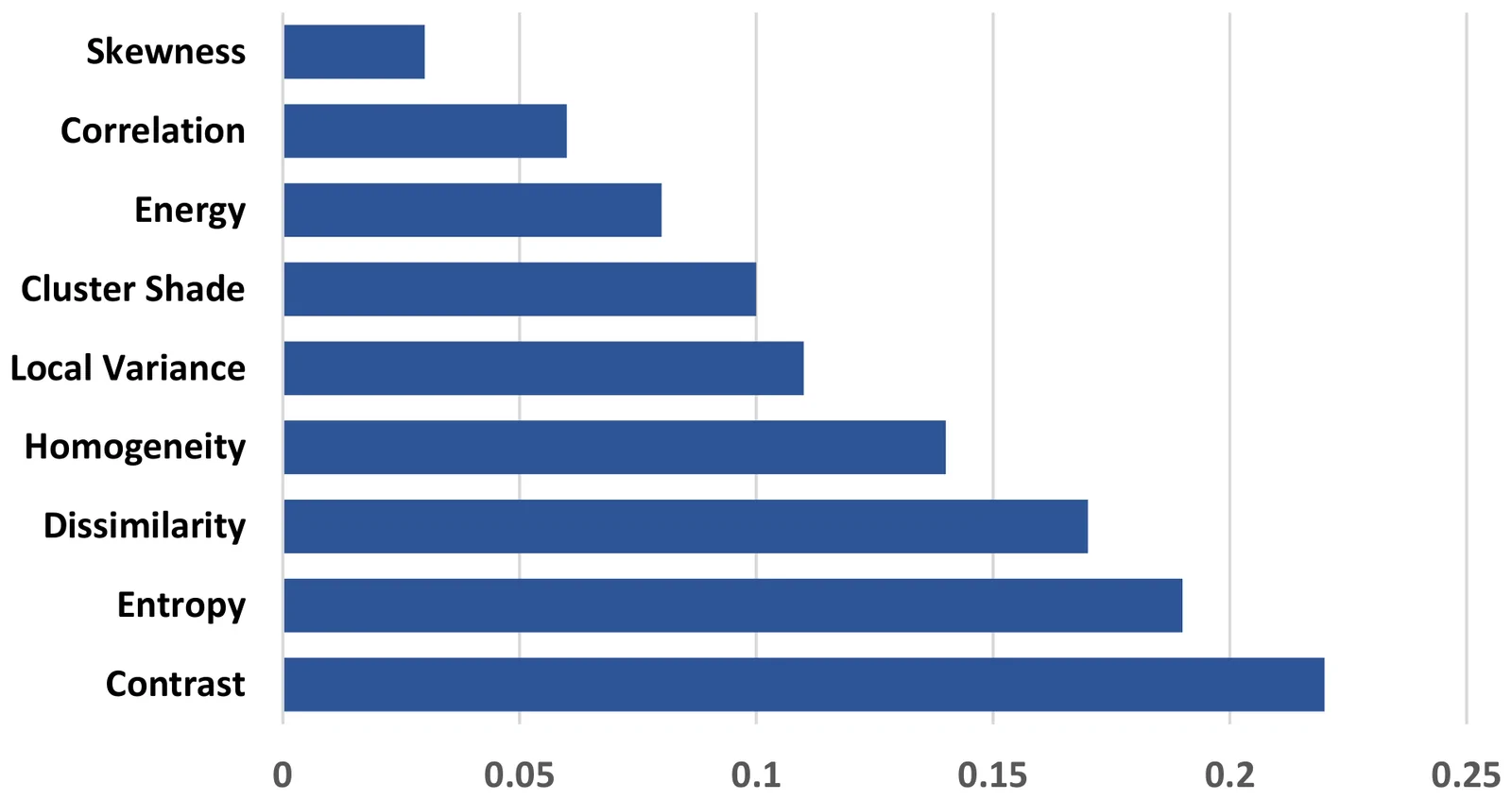
Permutation feature importance (PFI) of GLCM texture descriptors derived from laser speckle images.

To improve the interpretability of the developed ML framework, permutation feature importance (PFI) analysis was performed using the best-performing ensemble classifiers. The relative importance of the extracted GLCM descriptors is presented in Figure 5. The analysis revealed that contrast, entropy, and dissimilarity were the most influential features contributing to drought stress classification, followed by homogeneity, cluster shade, and local variance. In contrast, energy, correlation, and skewness exhibited comparatively lower importance. The consistency of the feature ranking across cross-validation folds indicates that the identified discriminative descriptors are stable and not dependent on a specific training–testing data partition.

These findings are physically meaningful because drought stress induces changes in leaf hydration status, internal cellular organization, and optical scattering properties, which directly influence the spatial heterogeneity of the speckle pattern. Features such as contrast, entropy, and dissimilarity are particularly sensitive to these changes and therefore contribute most strongly to model predictions. The obtained results further demonstrate that the proposed LSI-ML framework relies on physiologically relevant and interpretable texture descriptors rather than arbitrary image characteristics, thereby improving both the transparency and reproducibility of the classification process.

Table 5 shows the confusion matrix obtained for the six best-performing ML classifiers. It can be inferred from the table that XGBoost, RF, and decision trees contain a lower number of false positives and false negatives. Furthermore, exhaustive evaluation of ML models was performed by calculating various metrics, which are detailed in subsequent sections.

Performance metrics for classification results obtained for all ML models are presented in Table 6. Final analysis shows that XGBoost, RF, and decision trees performed quite well among all classifiers, with higher accuracy values of 96.67%, 95.56%, and 95.56%, respectively. Moreover, we also obtained higher precision, recall, and F1 score for these classifiers. These models presented higher recall values (96.67%, 95.56%, and 95.56%), which indicates that these classifiers correctly identified most observations in each of the three classes. It also implies that the model is capable of correctly identifying observations that actually belong to the early-stress or stressed class. The high specificity value denotes that these models are also capable of correctly rejecting observations that do not belong to a given class. GB, KNN, and SVM classifiers followed with classification accuracy (93.33%, 93.33%, and 88.89%, respectively) and comparatively lower precision, recall, F1 score, and specificity.

To check that the results do not depend on a single partition of the plants, stratified group-based 10-fold cross-validation was performed with plant identity as the grouping variable (Table 7). All feature vectors of a plant were kept in the same fold, and identical folds were used for all classifiers. The mean cross-validated accuracies ranged from 88.56% to 95.11%, with fold-to-fold standard deviations of 1.67–3.18 percentage points. XGBoost gave the highest mean accuracy, and the support vector machine the lowest, as on the independent test set. The cross-validated accuracies were within about 3 percentage points of the independent test-set accuracy in Table 6.

**Figure 6:**
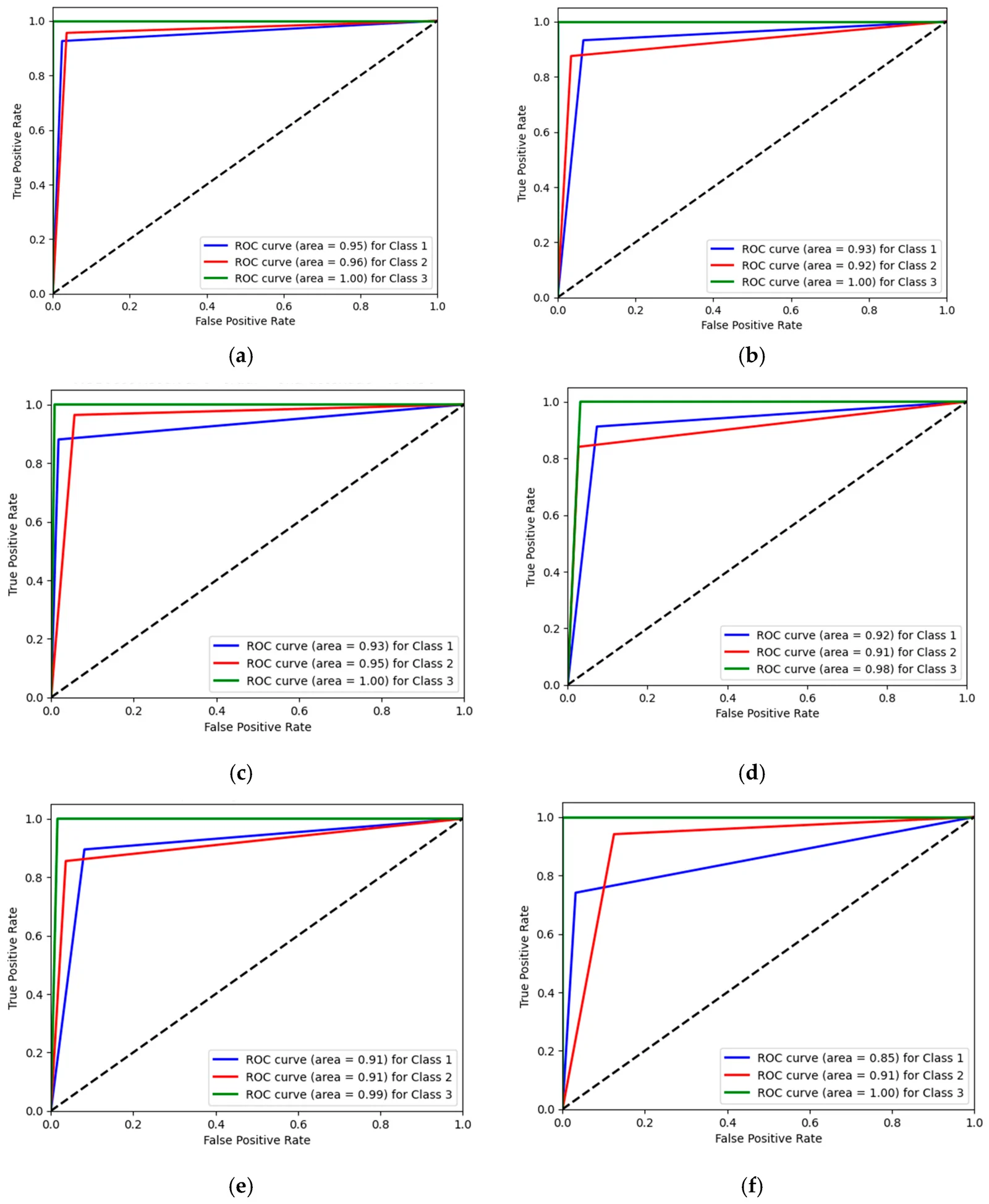
Receiver operating characteristics for all machine learning (ML) models: (**a**) random forest, (**b**) gradient boosting, (**c**) XGBoost, (**d**) decision trees, (**e**) K-nearest neighbors (KNN), and (**f**) support vector machine (SVM). The black dashed diagonal line represents a random classifier (AUC = 0.5).

Figure 6a–f depicts the ROC curves comparing predictive capability of various ML models. The ROC curve provides the performance of ML classifiers across a spectrum of TPR versus FPR. The diagonal line on an ROC curve represents the performance of the ML model, which yields positive or negative results dissimilar to the actual class. It means the dotted diagonal black line in Figure 6 represents the ROC curve for a purely random classifier, indicating inaccurate prediction. Therefore, this diagonal line serves as a benchmark for evaluating the performance of various ML classifiers. Ideally, the ROC curve for a good classifier must remain away from this diagonal line (i.e., toward the top-left corner of the curve). A quantitative measure to estimate the performance of the ML model by utilizing the ROC curve is the area under the ROC curve, termed ROC score. An AUC of 1 indicates perfect ranking of the evaluated samples, rather than necessarily zero errors at the selected classification threshold, whereas a random classifier scores 0.5. Figure 6a–f presents the ROC curve for prediction of drought stress in soybean plants. We have plotted the ROC curves for different classes and also calculated the class-wise ROC scores. This helps in identifying the ability of an ML model to classify a specific class, including early identification, as there were no visible symptoms of drought at this stage, and models can give wrong predictions. It can be inferred from the graph below that GB and RF classifier presented high values of AU-ROC scores for each class. As evident from the curves, the majority of the ML models reached an AUC of 1 for the stressed class, which indicates that these models ranked the stressed samples above the samples of the other classes. This does not imply the absence of misclassification at the selected classification threshold, for which the confusion matrices in Table 5 should be consulted. The stressed class is therefore well separable from the other classes. Moreover, for early-stress identification, these models presented a high AU-ROC score, reaching up to 0.96. This implies that the model has high classification performance for stress in early stages too. 

To assess robustness across genetic diversity, the dataset was purposefully curated by considering five soybean varieties, and all speckle images were pooled to create the final dataset for model development. The method also demonstrated its ability in the early-stage identification of drought stress. The dataset for the early-stress class contains speckle images for the samples, which have no specific visible symptoms of drought, as the speckle images were recorded for just five days after withholding irrigation in pots. Therefore, in this brief duration, there were no visual morphological changes (such as leaf wilting or alterations in leaf color) associated with drought. This capability arises from the photonics-based sensing principle of LSI, which takes into account the biological phenomena occurring within plant leaves rather than external leaf morphology. Consequently, the sensing approach delivers consistent performance across varieties. Moreover, it is feasible to identify drought stress conditions in plants even when no visible symptoms are apparent, as the sensor does not depend on the morphology of the plants. This robustness allows for consistent performance across different soybean varieties and facilitates drought identification even before visible signs of drought stress are on the plant leaf.

**Figure 7:**
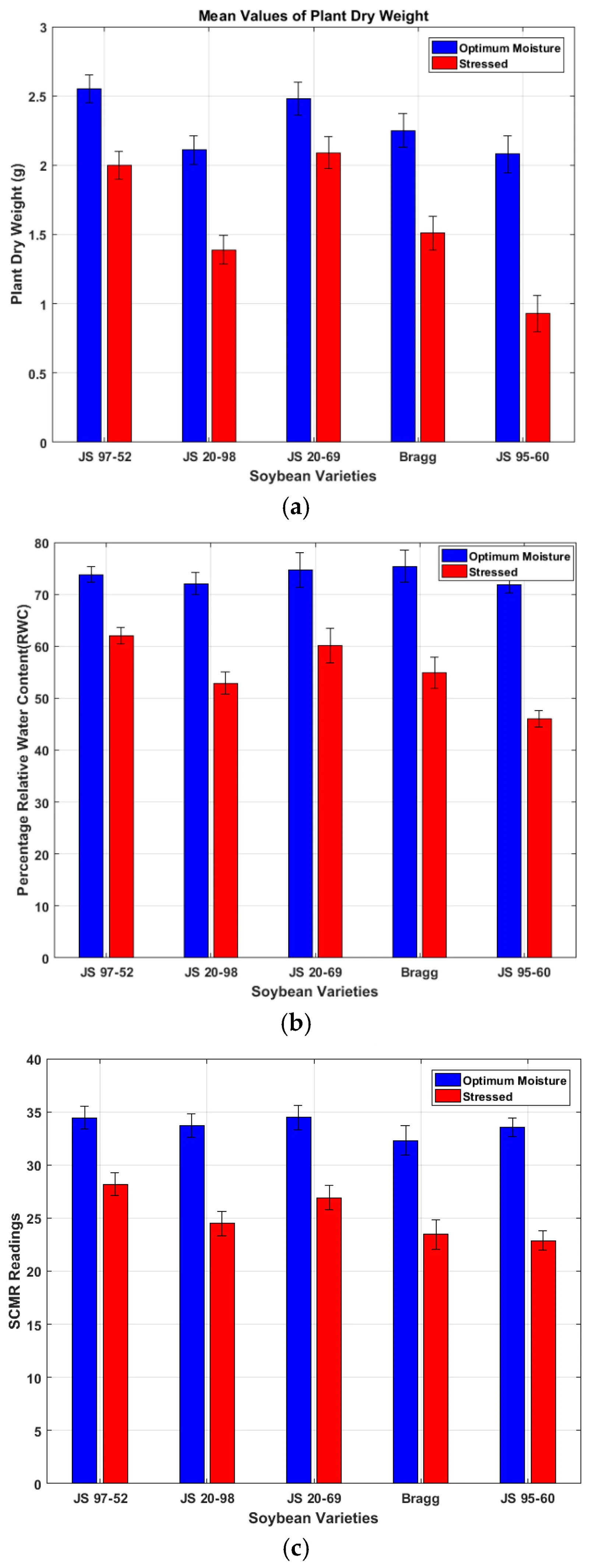
Variations in (**a**) plant dry weight (PDW), (**b**) relative water content (RWC), and (**c**) SPAD chlorophyll meter reading (SCMR) values for optimum moisture and stressed plants.

Figure 7 shows a comparison of morpho-physiological parameters, including mean values of PDW (in grams per plant), RWC, and SCMR values for both optimum moisture and stressed plants. The analysis revealed that the values of PDW, RWC, and SCMRs were significantly (*p* < 0.01) influenced by drought conditions across all varieties. However, in control conditions, varieties did not show significant variation (*p* > 0.05) for the traits except PDW, while in stress conditions, genotypes showed significant variations (*p* < 0.01), and JS 97-52 and JS 95-60 were found to be contrasting genotypes after the induction of stress. The details of the statistical analysis are presented in Table S1 of the Supplementary Material. It can be inferred from the plots below that the mean values of PDW, RWC, and the SCMRs were lower for stressed plants as compared to the controlled ones. Under control conditions (optimum moisture), the PDW varied from 2.08 g/plant to 2.55 g/plant, with a mean value of 2.37 g/plant. In contrast, under drought conditions, the range of PDW varied from 0.93 g/plant to 2.00 g/plant with average value 1.59 g/plant, showing lower biomass accumulation in drought conditions across all these varieties. Similarly, RWC values ranged from 71.81% to 73.81% for plants belonging to the optimum moisture class. The overall mean RWC for optimum moisture is relatively high at 73.55%, reflecting the well-hydrated state of the plants. Based on the morpho-physiological dataset, the following quantitative trends were observed: Under optimum moisture conditions, the RWC across soybean varieties ranged from 71.81% to 73.81%, with an overall mean of 73.55%, indicating fully hydrated leaf tissue. However, during the early-stress period (15–20 days), RWC showed a clear and statistically significant decline, with mean values reduced to approximately 62–65%, reflecting the onset of tissue dehydration and partial loss of cell turgor, despite the absence of visible wilting. A similar statistically significant trend was observed for SCMRs. SCMR values ranged from 33.55 to 34.45 (mean ≈ 33.69), corresponding to healthy chlorophyll content. Moreover, for the stressed class, SCMR values further decreased to 22.89–28.19 (mean ≈ 25.19), consistent with advanced drought stress. Statistical analysis using analysis of variance (ANOVA) confirmed that the reductions in both the RWC and SCMR during the early-stress stage are significant (*p* < 0.01) when compared to optimum moisture conditions. Under stressed conditions, the RWC declined sharply to values ranging from 45.98% to 62.08%, with an overall mean of 55.18%, confirming severe water deficit. Under drought stress, the RWC value decreased and ranges from 45.98% to 62.08%, with the overall mean value decreasing to 55.18%. This indicates a significant reduction in plant hydration levels due to water scarcity. SCMR values ranged from 33.55 to 34.45, with an average value of 33.69 for the plants with optimum moisture content. On the other hand, plants experiencing drought stress exhibited lower SCMR values ranging from 22.89 to 28.19, with an average value of 25.19. The higher mean SCMR values under optimum moisture conditions reflect sufficient chlorophyll content and efficient photosynthesis in well-hydrated plants. Conversely, due to water scarcity, the mean SCMR values decreased for all the varieties in stressed plants.

### 4.2. Correlation Analysis of GLCM Features and Physiological Parameters

To evaluate the physiological significance of the extracted speckle features, correlation analysis was performed between GLCM-derived parameters and independently measured physiological characteristics (RWC and SCMR). These parameters are well-established indicators of plant water status, cell turgor, and photosynthetic capacity under drought stress. Because the relationships between the speckle descriptors and the physiological indicators were not assumed to be linear, Spearman’s rank correlation [26] was employed.

Spearman’s rank correlation was computed using individual plant-level paired observations pooled across the three drought conditions (*n* = 450 plant condition observations from 300 plants), obtained from the plants selected for physiological measurements (30 plants per genotype in each group; 150 plants per group), in which each plant’s GLCM feature vector for a given condition was matched to its corresponding RWC and SCMR measurement. Each plant contributed one feature vector per condition; therefore, no additional averaging was required. Also, each plant condition observation was matched to the plant’s own RWC and SCMR measurement. Because early-stress and stressed observations were obtained from the same Group 2 plants, 150 out of the 300 plants contribute two observations each to this pooled set, so the 450 coefficients are reported descriptively rather than as the basis for significance testing. Statistical inference was instead based on a sensitivity analysis using one observation per plant (*n* = 300; all optimum moisture plants and the stressed observation of each Group 2 plant), in which every plant contributes exactly one independent observation. The resulting coefficients and Benjamini–Hochberg-adjusted *p*-values are reported in Table 8. The coefficients describe plant-level association; plants sharing a pot are not fully independent.

The correlation analysis revealed statistically significant associations between several GLCM descriptors and physiological indicators of drought stress. Contrast and dissimilarity, which decreased consistently from optimum moisture to early-stress and stressed conditions (Table 4), showed positive correlations with RWC and SCMRs. Correlation and cluster shade, which increased consistently across the three conditions, showed negative correlations. Energy, homogeneity, and skewness followed a non-monotonic pattern, decreasing slightly from optimum moisture to early stress and increasing under stressed conditions; their negative correlations with RWC and SCMRs (adjusted *p* < 0.001) therefore mainly reflect their higher values in the stressed class rather than a steady increase with drought severity. Entropy and local variance showed no significant association with either indicator. These results indicate a quantitative relationship between speckle texture variations and plant physiological status, suggesting that the proposed approach is biologically interpretable and physiologically relevant. The observed correlations indicate that several of the extracted GLCM features are sensitive to drought-induced physiological changes and are associated with plant hydration and chlorophyll status. When correlations were computed separately within each drought class (*n* = 150 per class), none was statistically significant, indicating that the descriptors primarily reflect drought-stage-dependent changes rather than plant-to-plant variation in water status within a given stage. Importantly, the speckle features exhibited distinct values across the optimum moisture, early-stress, and stressed classes, demonstrating sensitivity to drought progression prior to the appearance of visible symptoms.

Contrast and dissimilarity were positively associated with RWC and SCMRs, while correlation, skewness, cluster shade, homogeneity, and energy were negatively associated (adjusted *p* < 0.001); for energy, homogeneity, and skewness, this association reflects higher values under stress rather than a monotonic trend. Entropy and local variance showed no significant association, indicating that not all texture descriptors contribute equally to physiological interpretability and that contrast, dissimilarity, and correlation are the most robust indicators of drought-induced speckle texture change. The texture descriptors reflect drought-stage-dependent changes that accompany the declines in RWC and SCMRs, rather than plant-to-plant variation in water status within a given stage.

### 4.3. Analysis of Differences in Genetic Tolerance of a Given Variety Against Drought Stress

**Figure 8:**
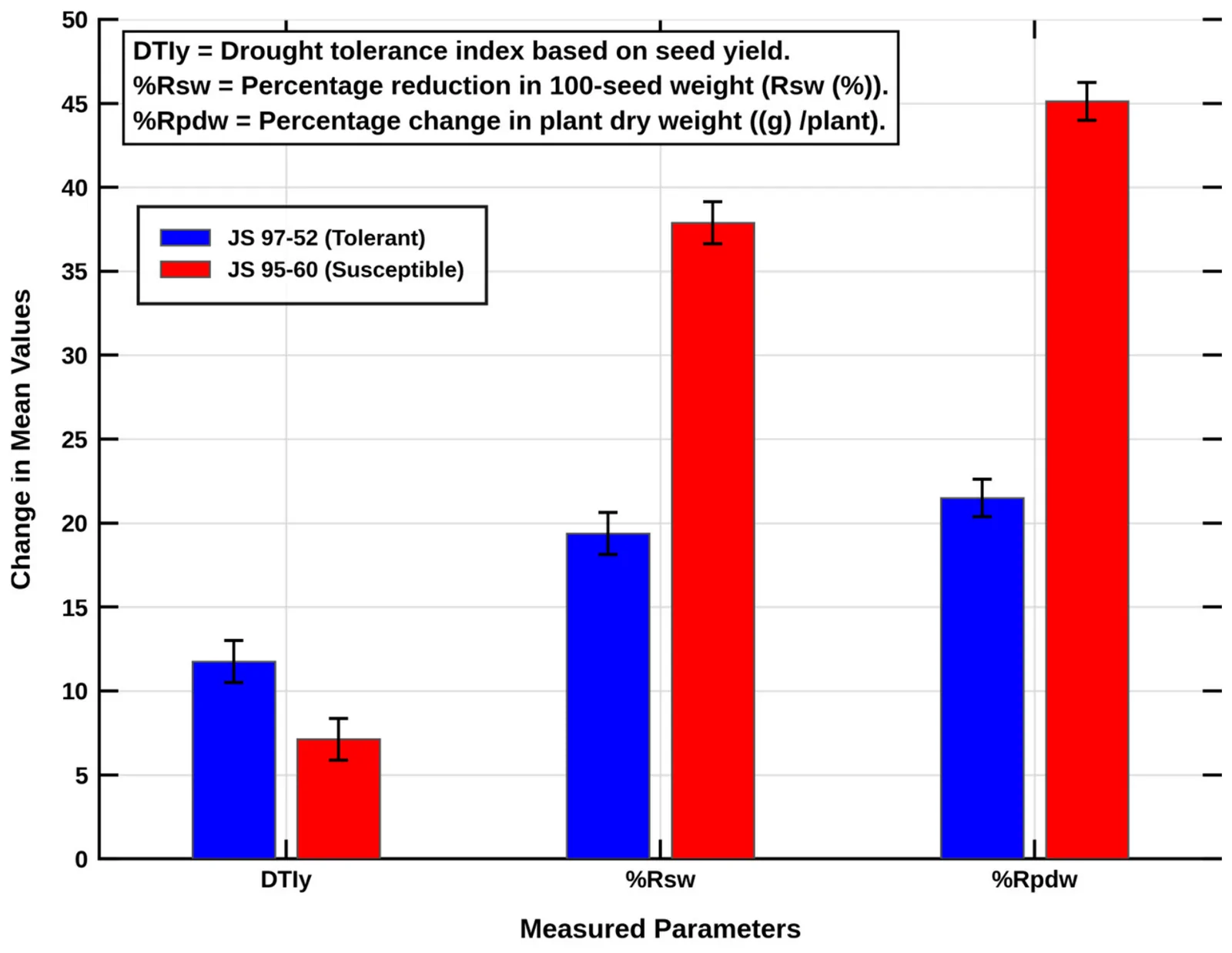
Variations in drought tolerance index (DTIy), percentage reduction in 100-seed weight (%Rsw), and percentage change in plant dry weight (g)/plant (%Rpdw) for drought-tolerant and -susceptible varieties.

The development of drought-tolerant varieties is one of the most effective, reliable, and economic methods to manage drought stress in crops. One of the objectives of this investigation is to analyze the capability of the proposed technique to identify the drought tolerance of different varieties towards drought, even in the early stages of drought. To investigate this, two distinct soybean varieties (JS 97-52 (tolerant) and JS 95-60 (susceptible)) with known differences in drought tolerance were used. Genetic tolerance of given varieties was revalidated by calculating various morpho-physiological parameters, including the drought tolerance index (DTIy) (based on seed yield), percentage reduction in 100-seed weight (%Rsw), and percentage change in PDW (Rpdw) ((g)/plant) during field studies in real-time situations. As shown in Figure 8, the drought-tolerant genotype (JS 97-52) exhibits superior performance as compared to the susceptible genotype (JS 95-60) and presents a significant difference in the mean values (*p* < 0.01). The detail of the statistical analysis is presented in Table S2 of the Supplementary Materials. JS 97-52 exhibited a high DTIy value of 11.17, which was significantly (*p* < 0.01) higher than that of the drought-susceptible genotype JS 95-60 (6.92). The percentage reduction in 100-seed weight (Rsw (%)) was also significantly (*p* < 0.01) influenced by genotypes under drought conditions. JS 97-52 displayed the lowest Rsw value of 20.55%, which was significantly (*p* < 0.01) lower than that of the drought-susceptible genotype JS 95-60 (39.79%). Similarly, the percentage change in plant dry weight ((g)/plant (%Rpdw)) was also significantly (*p* < 0.01) affected under drought conditions. JS 97-52 exhibited a minimum Rpdw value of 21.36%, which was significantly lower than that of the drought-susceptible genotype JS 95-60 (45.11%). 

To differentiate the drought tolerance of a given variety in the early stages (before specific symptoms became visible), several ML algorithms were implemented, and the dataset was divided into two classes: tolerant and susceptible. A total of 180 feature vectors per class (90 plants per genotype, 2 feature vectors per plant) were used to train, validate, and test the ML models, with a plant-exclusive 60/20/20 partition (108, 36, and 36 plants) yielding an independent test set of 72 feature vectors from 36 plants (36 per class). Table 9 and Table 10 show the confusion matrix and performance indicators for the six best-performing ML models while classifying the tolerance of given varieties against drought stress. To detect the drought tolerance capability of a given variety, in its early stages of growth, the dataset was recorded for five consecutive days just after the water supply was stopped. As shown in Table 10, most of the models presented high accuracy while classifying speckle images of soybean plants for both tolerant and susceptible varieties. Final analysis shows that SVM, MLP, and KNN performed quite well among all classifiers, with the highest accuracy (95.83% each), followed by RF (94.44%). Across most classifiers, recall was higher for the susceptible class than for the tolerant class (e.g., SVM: 100.00% vs. 91.67%), indicating that misclassifications occurred mainly among tolerant samples. It should be noted that in the present study, the tolerant class consisted exclusively of genotype JS 97-52 and the susceptible class exclusively of genotype JS 95-60; therefore, the reported classification accuracy reflects the ability of the model to distinguish these two specific, contrasting genotypes examined in this study, rather than a generalized drought tolerance signature applicable across genotypes. Speckle-derived features were found to be sensitive to the differing drought responses of these two genotypes, and validation using multiple tolerant and susceptible genotypes with a genotype-independent evaluation scheme (e.g., leave-one-genotype-out cross-validation) is identified as necessary future work before this capability can be generalized. Moreover, we have also obtained higher values of precision, recall, and F1 score for these classifiers on the independent test set. Most models presented high values of other performance parameters for classifying drought-tolerant and -susceptible varieties even in early stages of drought stress, whereas logistic regression showed the lowest performance (88.89%).

The same stratified group-based 10-fold cross-validation was applied to the classification of the tolerant and susceptible genotypes (Table 11). The mean cross-validated accuracies ranged from 87.78% to 94.72%, with standard deviations of 2.46–5.56 percentage points. The variability across folds was larger than in Experiment 1 because each fold contained only about 18 plants (36 feature vectors), so one misclassified vector changes the fold accuracy by higher percentage points. Multilayer perceptron, K-nearest neighbors, and support vector machine gave the highest mean accuracies and logistic regression the lowest, as shown in Table 10. The cross-validated accuracies were within about 2 percentage points of the independent test-set accuracies.

**Figure 9:**
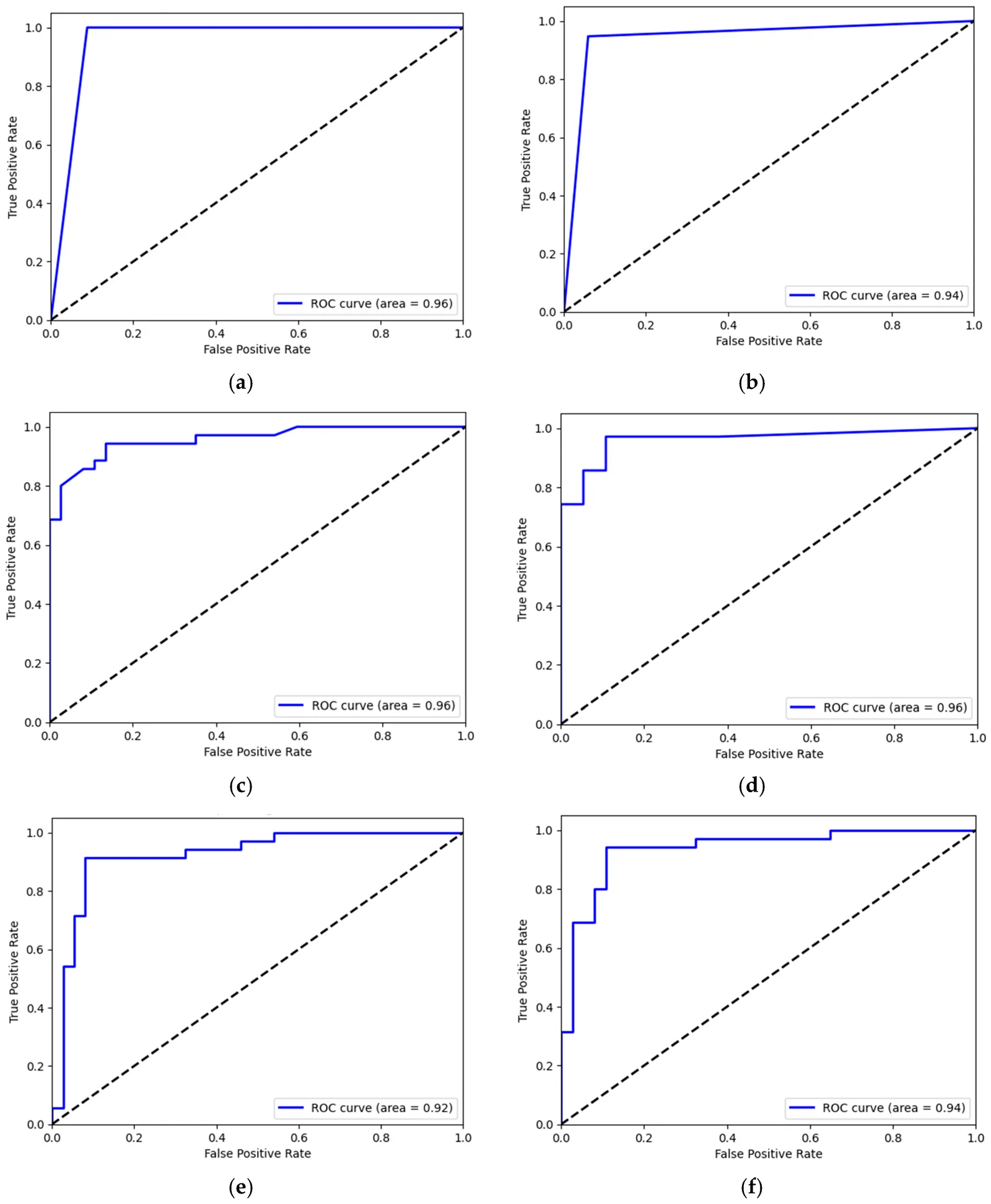
Receiver operating characteristics for all the machine learning (ML) models: (**a**) support vector machine (SVM), (**b**) multilayer perceptron (MLP), (**c**) random forest, (**d**) K-nearest neighbors (KNN), (**e**) gradient boosting, and (**f**) logistic regression (LR). The black dashed diagonal line represents a random classifier (AUC = 0.5).

Similar to the above section, the ROC curve and its associated AU-ROC score for various ML models are given in Figure 9a–f. In the prediction of drought stress in soybean plants, the RF classifier presented high values of AU-ROC. This suggests that the models are capable of classifying plants based on their genetic tolerance towards drought. The ROC curves for the SVM and MLP classifiers were away from the diagonal line and resulted in higher AU-ROC scores (0.96 and 0.94, respectively). These above analyses indicate that JS 97-52 is a drought-tolerant variety and may be the preferable choice for cultivation in regions prone to water scarcity. This genotype is also being used as a potential donor in breeding programs to develop climate-smart soybean cultivars in India [27]. These findings underscore the importance of selecting drought-tolerant varieties to enhance agricultural resilience and productivity due to water scarcity.

The results obtained in this study indicate that LSI, when integrated with ML, can be used to monitor drought stress in soybean plants under controlled laboratory conditions. By capturing biological signatures of plants through LSI and translating them into actionable insights via ML, the proposed sensor achieves early-stage identification of drought stress, classification of severity, and assessment of stress tolerance capabilities for different varieties. In this study, a single LSI setup combined with ML was used for drought severity classification, early-stage detection, and discrimination of two contrasting genotypes, using a non-contact measurement that does not depend on visible symptoms. These measurements are relevant for proactive management of drought stress. A direct comparison with other sensing techniques was not part of this study.

The objective of the present study is not to establish the superiority of laser speckle imaging over existing plant phenotyping techniques but rather to evaluate its feasibility, sensitivity, and physiological relevance for drought stress detection. Well-established approaches such as hyperspectral imaging, multispectral imaging, chlorophyll fluorescence imaging, and thermal imaging provide valuable biochemical, physiological, and thermal information and have been widely employed for plant stress assessment [28,29]. However, these modalities often require complex instrumentation, controlled illumination conditions, calibration procedures, and high-dimensional data processing pipelines. It is also important to note that many studies that established hyperspectral imaging, chlorophyll fluorescence imaging, and thermal imaging as useful tools for plant stress detection primarily focused on validating the sensing capability of the individual modality using physiological measurements and stress indicators, rather than performing direct comparisons with all available sensing methods [28,29,30]. Similarly, the present work focuses on validating the physiological relevance and sensing capability of LSI through independently measured indicators such as relative water content (RWC) and SPAD chlorophyll meter readings (SCMRs).

In contrast, LSI relies on coherent light scattering and is particularly sensitive to drought-induced microstructural and hydration-dependent changes in plant tissues. Previous studies have reported the advantages of coherent optical sensing approaches in terms of hardware simplicity, computational efficiency, and sensitivity to structural changes [28,29,30]. These reported advantages are not claimed as novel in the present work but rather form the conceptual motivation for exploring LSI as an early-warning sensing system in plant drought stress studies. Traditional destructive measurements (e.g., gravimetric and biochemical) are labor-intensive and unsuitable for repeated high-throughput screening. Relative to established optical techniques such as hyperspectral imaging and chlorophyll fluorescence, which provide biochemical and photosynthetic information, LSI requires lower hardware cost and complexity, reduced calibration burden, and modest computational demands, making it more practical for rapid early-stage screening and deployment in resource-constrained settings. In contrast, RGB imaging with deep learning generally excels at recognizing visible morphology, color changes, and semantic patterns but lacks direct sensitivity to pre-symptomatic hydration and microstructural processes. These approaches also require large, labeled datasets and substantial computational resources and often yield less physiologically interpretable features. Speckle-derived GLCM texture descriptors provide physically grounded, data-efficient, and interpretable representations of coherent light–tissue interactions, aligning well with the goal of early drought stress detection under controlled acquisition conditions. While the LSI-based framework offers potential advantages (e.g., non-contact, independent of visible symptoms) in practicality and early-stage sensitivity, it remains dependent on controlled measurement conditions and would benefit from future work on domain transfer across cultivars, growth stages, and environments, as well as lightweight multimodal fusion with targeted physiological indices and physics-informed learning approaches to further strengthen its robustness and translational potential.

These outcomes collectively underscore the potential of our proposed technique to offer valuable insights that can contribute to the efficient management of drought stress in plants. The proposed LSI approach is inherently one of the more suitable optical techniques for field adaptation and miniaturization in future. Because LSI relies on narrow-band coherent illumination, the impact of ambient light could potentially be mitigated using wavelength-matched optical filters, simple shielding, or synchronized acquisition, which are commonly employed in outdoor optical sensing; this remains to be validated under field conditions. The use of single-frame acquisition with short exposure times reduces sensitivity to wind-induced motion and vegetal movement, making the method more robust than time-averaged or dynamic speckle approaches under outdoor conditions. Furthermore, the study was also supported by field data, which serves as an essential benchmark for assessing the influence of drought stress on soybean growth and biomass production. We also believe that the proposed technique may be useful to farmers and personnel associated with agricultural research and policy, directly or indirectly, for arriving at decisions regarding drought stress severity. By using such a tool, accurate decisions for better plant protection and drought management can be made.

## 5. Conclusions

The results obtained in this study indicate that LSI, as a photonics-based sensing technique in conjunction with ML, can be a promising tool for monitoring drought stress in plants under controlled laboratory conditions. LSI enables non-contact detection of physiological changes to trace the progression of drought without relying on visible symptoms. XGBoost achieved the highest classification accuracy (96.67%) for drought stress identification, including the early-stress stage. The proposed technique also discriminated the two contrasting genotypes examined for drought tolerance in this study (95.83%). However, as each tolerance class was represented by a single genotype, this result reflects genotype discrimination under the tested conditions rather than a generalized drought tolerance signature. In addition, the proposed method characterizes speckle-based signatures associated with drought and its related parameters. The extracted speckle descriptors were significantly correlated with RWC and SCMRs, which supports their physiological relevance and opens avenues for understanding the physiological as well as biological responses of soybean plants under drought stress. These outcomes indicate that the proposed technique can provide information relevant to the management of drought stress in plants. Furthermore, the study was also supported by field data, which serve as a reference for assessing the influence of drought stress on soybean growth and biomass production. Nevertheless, real-time operation and field deployment were not evaluated in the present study, and a direct comparison with other sensing techniques was not part of this work. Future work will focus on validation under variable illumination, plant motion, and changing environmental conditions and across multiple tolerant and susceptible genotypes using a genotype-independent evaluation scheme. Following further validation under field conditions, the proposed technique may be useful to farmers and personnel associated with agricultural research and policy for arriving at decisions regarding drought stress severity.

## Figures and Tables

**Table 1:** Dataset description for classification of drought stress.

Classes	Treatments	Varieties	Number of Speckle Images
Optimum Moisture (no drought stress in plants)	Provided water at every stage of plant development	JS 97-52	600
		JS 20-69	600
		JS 20-98	600
		Bragg	600
		JS 95-60	600
Early Stress (no visible symptoms of drought stress)	Watering is stopped after 15 days of sowing	JS 97-52	600
		JS 20-69	600
		JS 20-98	600
		Bragg	600
		JS 95-60	600
Stressed (visible symptoms of drought stress are available on plant leaves)	Watering is stopped after 15 days of sowing	JS 97-52	600
		JS 20-69	600
		JS 20-98	600
		Bragg	600
		JS 95-60	600

**Table 2:** Dataset description for identification of drought-tolerant and -susceptible varieties.

Classes	Treatments	Varieties	Number of Speckle Images
Tolerant	Watering is stopped after 15 days of sowing	JS 97-52	1800
Susceptible	Watering is stopped after 15 days of sowing	JS 95-60	1800

**Table 3:** Hyperparameter search space and final model configuration for the classifiers used in Experiments 1 and 2.

Classifier	Hyperparameter	Search Space	Selected Value
Gradient Boosting	$n_{estimator}$	{100, 200, 300}	200
	Learning rate	{0.01, 0.05, 0.10}	0.05
	Maximum depth	{3, 5, 7}	5
Random Forest	*n_estimators_*	{100, 200, 300}	200
	Maximum depth	{None, 5, 10, 15}	10
	Minimum samples split	{2, 5, 10}	2
XGBoost	*n_estimators_*	{100, 200, 300}	200
	Learning rate	{0.01, 0.05, 0.10}	0.10
	Maximum depth	{3, 5, 7}	5
	Subsample ratio	{0.8, 1.0}	0.8
Decision Tree	Maximum depth	{3, 5, 10, None}	10
	Minimum samples split	{2, 5, 10}	2
	Criterion	{gini, entropy}	gini
K-Nearest Neighbors	*k*	{3, 5, 7, 9, 11}	5
	Weighting	{uniform, distance}	distance
	Distance metric	{euclidean, manhattan}	euclidean
Support Vector Machine	*C*	{0.1, 1, 10, 100}	10
	Kernel	{linear, RBF}	RBF
	γ	{scale, 0.01, 0.1, 1}	scale
Logistic Regression	*C*	{0.01, 0.1, 1, 10, 100}	1
	Penalty	{L2}	L2
	Solver	{lbfgs, liblinear}	lbfgs
Multilayer Perceptron	Hidden-layer size	{(50), (100), (50,50)}	(100)
	Learning rate	{0.001, 0.01}	0.001
	Regularization parameter (α)	{1 × 10^−4^, 1 × 10^−3^}	1 × 10^−4^

**Table 4:** Summary of extracted GLCM texture features under different drought conditions, including optimum moisture, early stress, and stressed.

S. No.	Feature	Optimum Moisture (Mean ± SD)	Early Stress (Mean ± SD)	Stressed (Mean ± SD)
1	Contrast	1436.86 ± 321.30	1248.16 ± 261.46	628.50 ± 286.11
2	Dissimilarity	16.16 ± 2.59	15.76 ± 2.38	10.19 ± 3.02
3	Energy	0.100 ± 0.035	0.075 ± 0.018	0.129 ± 0.050
4	Homogeneity	0.594 ± 0.032	0.580 ± 0.010	0.628 ± 0.034
5	Correlation	0.313 ± 0.074	0.534 ± 0.090	0.928 ± 0.054
6	Entropy	1.001 ± 0.220	0.966 ± 0.055	1.043 ± 0.158
7	Cluster Shade	−6.63 × 10^7^ ± 1.67 × 10^7^	−5.91 × 10^7^ ± 1.60 × 10^7^	−4.36 × 10^7^ ± 2.13 × 10^7^
8	Skewness	0.865 ± 0.018	0.849 ± 0.021	0.899 ± 0.031
9	Local Variance	1.093 ± 0.563	1.007 ± 0.330	1.032 ± 0.644

**Table 5:** Confusion matrices of the best-performing classifiers for drought stress classification (Experiment 1; independent test set, *n* = 180).

Classifier Model			Predicted Class		
			Optimum Moisture	Early Stress	Stressed
Gradient boosting	**Actual Class**	Optimum Moisture	54	5	1
		Early Stress	6	54	0
		Stressed	0	0	60
Random forest		Optimum Moisture	55	5	0
		Early Stress	2	58	0
		Stressed	1	0	59
XGBoost		Optimum Moisture	59	1	0
		Early Stress	2	56	2
		Stressed	0	1	59
Decision trees		Optimum Moisture	55	3	2
		Early Stress	2	58	0
		Stressed	1	0	59
K-nearest neighbors (KNN)		Optimum Moisture	54	4	2
		Early Stress	1	59	0
		Stressed	2	3	55
Support vector machine (SVM)		Optimum Moisture	46	14	0
		Early Stress	4	56	0
		Stressed	1	1	58

**Table 6:** Performance parameters obtained for different classifiers for drought stress classification.

Classifier/Performance Matrix (%)	Accuracy	Macro Precision	Macro Recall	Macro F1 Score	Macro Specificity
XGBoost	96.67	96.66	96.67	96.65	98.33
Random forest	95.56	95.63	95.56	95.56	97.78
Decision trees	95.56	95.54	95.56	95.54	97.78
Gradient boosting	93.33	93.30	93.33	93.31	96.67
K-nearest neighbor	93.33	93.54	93.33	93.33	96.67
Support vector machine	88.89	89.69	88.89	88.89	94.44

**Table 7:** Stratified group-based 10-fold cross-validation accuracy for drought stress classification (Experiment 1; plant identity as grouping variable; identical folds for all classifiers) compared with the independent test-set accuracy in Table 6. CV: cross-validation; SD: standard deviation across the 10 folds.

Classifier	CV Mean ± SD (%)	CV Min–Max (%)
XGBoost	95.11 ± 1.67	93.33–98.89
Random forest	93.33 ± 2.46	88.89–96.67
Decision trees	92.89 ± 2.68	87.78–95.56
Gradient boosting	91.44 ± 2.29	87.78–95.56
K-nearest neighbors	92.44 ± 3.18	87.78–97.78
Support vector machine	88.56 ± 2.35	84.44–92.22

**Table 8:** Spearman rank correlations (ρ) between texture descriptors and physiological indicators (RWC and SCMR), computed from plant-level paired observations. ρ values at *n* = 450 are descriptive (pooled across all three conditions); ρ values and adjusted *p*-values at *n* = 300 are the basis for statistical significance, since every plant contributes exactly one observation” (the data are from a greenhouse, not the field) in this set.

S. No.	Texture Descriptor	ρ RWC (*n* = 450)	ρ RWC (*n* = 300)	Adj. *p* RWC (*n* = 300)	ρ SCMR (*n* = 450)	ρ SCMR (*n* = 300)	Adj. *p* SCMR (*n* = 300)
1	Contrast	0.699	0.720	<0.001	0.711	0.722	<0.001
2	Dissimilarity	0.608	0.685	<0.001	0.605	0.675	<0.001
3	Energy	−0.235	−0.327	<0.001	−0.218	−0.261	<0.001
4	Homogeneity	−0.367	−0.419	<0.001	−0.340	−0.363	<0.001
5	Correlation	−0.868	−0.734	<0.001	−0.881	−0.752	<0.001
6	Entropy	−0.112	−0.096	0.107	−0.107	−0.108	0.071
7	Cluster Shade	−0.410	−0.438	<0.001	−0.418	−0.434	<0.001
8	Skewness	−0.367	−0.471	<0.001	−0.377	−0.467	<0.001
9	Local Variance	0.044	0.022	0.698	0.018	0.004	0.939

**Table 9:** Confusion matrices of the best-performing classifiers for classification of tolerant (JS 97-52) and susceptible (JS 95-60) genotypes (Experiment 2; independent test set, *n* = 72).

Classifier			Predicted Class	
			Tolerant	Susceptible
Support vector machine	**Actual Class**	Tolerant	33	3
		Susceptible	0	36
Multilayer perceptron		Tolerant	34	2
		Susceptible	1	35
Random forest		Tolerant	33	3
		Susceptible	1	35
K-nearest neighbors		Tolerant	35	1
		Susceptible	2	34
Gradient boosting		Tolerant	32	4
		Susceptible	1	35
Logistic regression		Tolerant	30	6
		Susceptible	2	34

**Table 10:** Performance parameters obtained for different classifiers.

Classifier	Accuracy (%)	Macro Precision (%)	Macro Recall (%)	Macro F1 Score (%)	Tolerant Recall (%)	Susceptible Recall (%)
Support vector machine	95.83	96.15	95.83	95.83	91.67	100.00
Multilayer perceptron	95.83	95.87	95.83	95.83	94.44	97.22
K-nearest neighbors	95.83	95.87	95.83	95.83	97.22	94.44
Random forest	94.44	94.58	94.44	94.44	91.67	97.22
Gradient boosting	93.06	93.36	93.06	93.04	88.89	97.22
Logistic regression	88.89	89.38	88.89	88.85	83.33	94.44

**Table 11:** Stratified group-based 10-fold cross-validation accuracy for the classification of tolerant (JS 97-52) and susceptible (JS 95-60) genotypes (Experiment 2; plant identity as grouping variable; identical folds for all classifiers) compared with the independent test-set accuracy in Table 10. CV: cross-validation; SD: standard deviation across the 10 folds.

Classifier	CV Mean ± SD (%)	CV Min–Max (%)
Support vector machine	93.89 ± 3.46	88.89–96.17
Multilayer perceptron	94.72 ± 3.78	86.11–96.28
K-nearest neighbors	94.44 ± 2.61	88.89–95.62
Random forest	92.50 ± 5.56	83.33–97.47
Gradient boosting	92.22 ± 2.46	88.89–94.18
Logistic regression	87.78 ± 3.51	83.33–92.67

## Data Availability

Data and code are available from the corresponding author upon reasonable request.

## Supplementary Materials

The following supporting information can be downloaded at: https://www.mdpi.com/article/10.3390/s26216906/s1, Table S1: Effect of drought on morpho-physiological parameters of soybean plants; Table S2: Effect of drought on morpho-physiological parameters of tolerant and susceptible varieties.

## Abbreviations

The following abbreviations are used in this manuscript:

2D-GLCM	2D Gray Level Co-Occurrence Matrix
DL	Deep Learning
DT	Decision Trees
DTI	Drought Tolerance Indices
FPR	False Positive Rate
GB	Gradient Boosting
HIS	Hyperspectral Imaging
KNN	K-Nearest Neighbors
LR	Logistic Regression
LSD	Least Significant Difference
LSI	Laser Speckle Imaging
ML	Machine Learning
MLP	Multilayer Perceptron
NIRS	Near-Infrared Spectroscopy
PDW	Plant Dry Weight
RF	Random Forest
ROC	Receiver Operating Characteristic
RWC	Relative Water Content
SVM	Support Vector Machine
SCMR	SPAD Chlorophyll Meter Reading
TPR	True Positive Rate
