# Supplementary material for: Laser Speckle Imaging-Based Data-Driven Sensor for Comprehensive Monitoring of Drought Stress in Soybean Plants
Source: Sensors (Basel). 2026 Oct 31;26(21):6906. doi: 10.3390/s26216906 (PMC13766333; doi:10.3390/s26216906)
Supplement: Supplementary file 1 [file sensors-26-06906-s001.zip › sensors-4565336-supplementary.pdf]

The below tables provide statistical analysis performed on the experimental morpho-physiological parameters and other traits that were recorded from the plants of each pot as per standard protocol.

Table S1: Effect of drought on morpho-physiological parameters of soybean plants.

Genotypes	Plant dry weight((g)/plant		RWC/Relative water content (%)		SCMR/SPAD Reading	
	Control	Drought	Control	Drought	Control	Drought
JS 97-52	2.55ab	2.00a	73.81a	62.08a	34.45a	28.52a
JS 20-98	2.11ab	1.39ab	72.03a	52.89ab	33.70a	25.15ac
JS 20-69	2.84a	2.09a	74.73a	60.10a	34.46a	26.93ab
Bragg	2.25ab	1.51ab	75.40a	54.85ab	32.30a	23.47bc
JS 95-60	2.08b	0.93b	71.81a	45.98b	33.55a	22.89c
LSD	0.71	0.70	NS	13.41	NS	4.42

Table S2: Effect of drought on morpho-physiological parameters of tolerant and susceptible varieties.

Genotypes	Drought tolerance Index based on seed yield (DTIy)	Percentage reduction in 100 seed weight (Rsw (%)	Percentage change in plant dry weight ((g)/plant (% Rpdw)
**JS 97-52**	10.17a	20.55b	21.36c
**JS 20-98**	8.8bc	30.36ab	31.34b
**JS 20-69**	10.30ab	25.75b	28.11bc
**Bragg**	8.90bc	22.96b	28.57bc
**JS 95-60**	6.90c	39.79a	45.11a
**LSD**	0.24	12.77	7.91
